# Endodermal apoplastic barriers are linked to osmotic tolerance in meso-xerophytic grass *Elymus sibiricus*

**DOI:** 10.3389/fpls.2022.1007494

**Published:** 2022-09-23

**Authors:** Xin Liu, Ping Wang, Yongping An, Chun-Mei Wang, Yanbo Hao, Yue Zhou, Qingping Zhou, Pei Wang

**Affiliations:** ^1^ Sichuan Zoige Alpine Wetland Ecosystem National Observation and Research Station, Institute of Qinghai-Tibetan Plateau, Southwest Minzu University, Chengdu, China; ^2^ National Key Laboratory of Crop Genetic Improvement, Ministry of Agriculture Key Laboratory of Crop Ecophysiology and Farming System in the Middle Reaches of the Yangtze River, College of Plant Science and Technology, Huazhong Agricultural University, Wuhan, China; ^3^ Lanzhou Institute of Husbandry and Pharmaceutical Sciences, Chinese Academy of Agricultural Sciences, Lanzhou, China

**Keywords:** casparian strip, drought tolerance, *Elymus sibiricus*, endodermis, suberin

## Abstract

Drought is the most serious adversity faced by agriculture and animal husbandry industries. One strategy that plants use to adapt to water deficits is modifying the root growth and architecture. Root endodermis has cell walls reinforced with apoplastic barriers formed by the Casparian strip (CS) and suberin lamellae (SL) deposits, regulates radial nutrient transport and protects the vascular cylinder from abiotic threats. *Elymus sibiricus* is an economically important meso-xerophytic forage grass, characterized by high nutritional quality and strong environmental adaptability. The purpose of this study was to evaluate the drought tolerance of *E. sibiricus* genotypes and investigate the root structural adaptation mechanism of drought-tolerant genotypes’ responding to drought. Specifically, a drought tolerant (DT) and drought sensitive (DS) genotype were screened out from 52 *E. sibiricus* genotypes. DT showed less apoplastic bypass flow of water and solutes than DS under control conditions, as determined with a hydraulic conductivity measurement system and an apoplastic fluorescent tracer, specifically PTS trisodium-8-hydroxy-1,3,6-pyrenetrisulphonic acid (PTS). In addition, DT accumulated less Na, Mg, Mn, and Zn and more Ni, Cu, and Al than DS, regardless of osmotic stress. Further study showed more suberin deposition in DT than in DS, which could be induced by osmotic stress in both. Accordingly, the CS and SL were deposited closer to the root tip in DT than in DS. However, osmotic stress induced their deposition closer to the root tips in DS, while likely increasing the thickness of the CS and SL in DT. The stronger and earlier formation of endodermal barriers may determine the radial transport pathways of water and solutes, and contribute to balance growth and drought response in *E. sibiricus*. These results could help us better understand how altered endodermal apoplastic barriers in roots regulate water and mineral nutrient transport in plants that have adapted to drought environments. Moreover, the current findings will aid in improving future breeding programs to develop drought-tolerant grass or crop cultivars.

## Introduction

Drought is the most serious abiotic stress that restricts agriculture and animal husbandry development. Drought stress reduces turgor pressure, disrupts ion homeostasis, damages cell membrane systems, and inhibits photosynthesis, among other effects. Furthermore, it not only impedes plant growth and metabolism at different stages, but also affects crop yields and quality (Reddy et al., 2004; Passioura, 2007). Global losses in crop production due to drought have totaled ~US $30 billion over the past decade. With the rapid growth of the global population, high-yield plants that use water more efficiently than their modern counterparts are urgently required (Gupta et al., 2020). *Elymus sibiricus* (Siberian wild rye) is an economically important perennial allotetraploid, meso-xerophytic and high-yield forage grass, native to northern Asia. It is palatable, nutrient-rich, and easily digestible, which are conducive to the growth and reproduction of domestic animals. This grass is widely used in natural grasslands and cultivated pastures on the Qinghai-Tibet Plateau owing to good forage quality, adaptability and excellent cold and drought tolerance (Ma et al., 2012; Liu et al., 2016; Xie et al., 2017). However, little is known about drought resistance strategies in this species.

A water deficit is the main cause of drought stress. Therefore, maintaining sufficient water absorption and preventing water loss in water-deficient environments enhance the drought resistance of plants. Plant roots are the first organs that sense the soil water status, and manage water deficiencies (Steudle, 2000). Root systems determine the capacity of a plant to access soil water, and their morphology and architecture can influence adaptation to water-limited conditions (Singh et al., 2010). According to the composite transport model, water and solutes are transported rapidly from the rhizodermis to xylem vessels in the stele *via* cell-to-cell (symplastic and transcellular) and apoplastic pathways (Kreszies et al., 2018). The key factors of water movement through the cell-to-cell pathway and the underlying mechanism have been investigated. For example, aquaporins contribute to water flux, and hence, function in drought tolerance (Lian et al., 2006; Chaumont and Tyerman, 2014; Grondin et al., 2016). Although root apoplastic barriers are theoretically important for plant adaptation to environmental stresses such as drought, little is known about their function and regulation.

The root apoplastic barriers comprise the Casparian strip (CS) and suberin lamellae (SL) of the endodermis and periderm and provide the main resistance to radial water transport *via* transcellular and apoplastic pathways in roots, which likely plays a crucial role in drought tolerance (Steudle, 2000; Kreszies et al., 2019; de Silva et al., 2021). The CS is the localized impregnation of a primary cell that longitudinally encircles an endodermal cell and might prevent the free apoplastic bypass flow (apoplastic pathway) of solutes in the extracellular spaces between the cortex and inner vasculature (Enstone et al., 2002; Geldner, 2013). The SL is deposited on the entire inner face of the cell wall adjacent to the plasma membrane, and it might be instrumental in preventing the movement of water and solutes from apoplasts directly into the endodermal protoplasts (Schreiber et al., 1999; Ranathunge et al., 2005; Martinka et al., 2012). The SL is polymerized based on polyaliphatic and polyaromatic domains. The aliphatic monomers are mainly ω-hydroxy fatty acids (ω-OH acids) and α,ω-dicarboxylic fatty acids (α,ω-diacids), with some primary alcohols and unsubstituted fatty acids, whereas the aromatic components are mainly ferulic and coumaric acids (Bernards, 2002; Pollard et al., 2008; Vishwanath et al., 2015). The CS is comprised of lignin or a lignin-like polymer (Geldner, 2013). It can also include aliphatic suberin in some species (Zeier and Schreiber, 1997), although histochemical staining and chemical analysis have shown that the CS is comprised only of a lignin-like polymer without suberin in *Arabidopsis* (Naseer et al., 2012).

The deposition of CS and SL not only changes throughout plant development but can also be modulated by abiotic stresses, such as drought (Barberon, 2017; Doblas et al., 2017; Campilho et al., 2020; de Silva et al., 2021). The SL is enhanced by osmotic stress in the seminal roots of barley (*Hordeum vulgare*) (Kreszies et al., 2019). Moreover, suberin deposition is induced under water-deficit conditions in grapevine (*Vitis riparia*) fine roots (Zhang et al., 2020). The *Arabidopsis esb1* (*enhanced suberin 1*) mutant has higher water-use efficiency and lower transpiration rates than the wild-type, and this is associated with enhanced suberin deposition and ectopic lignin in roots (Baxter et al., 2009). The SL acts as a powerful barrier that prevents the uncontrolled backflow of water and solutes from the root to the medium and is important for *Arabidopsis* growth under drought and salt conditions (Wang et al., 2020a; de Silva et al., 2021). Natural variation in root suberization is associated with the local environment, especially edaphic water conditions, and the chemical composition, rather than only the amount of suberin, also plays a role in plant responses to drought and long-term adaptation to arid environments (Feng et al., 2022). Studies have also shown the role of the CS functions in the selective uptake of mineral nutrients and salinity tolerance of rice (*Oryza sativa*) and maize (*Zea mays*) (Krishnamurthy et al., 2011; Wang et al., 2019; Wang et al., 2022a; Wang et al., 2022b). Whereas the role of the CS as a barrier for water is poorly supported by functional evidence. Changes in root apoplastic barriers would alter water relationship and modulate drought tolerance in plants.

The total content and composition of suberin varies substantially between *Arabidopsis* and gramineous species, such as rice and barley. Various cultivars and growth conditions also contain different amounts of total suberin (Kreszies et al., 2018). Therefore, the study of root apoplastic barriers in gramineous grasses is necessary to understand the unique strategies used by grass and forage plants to adapt to environmental stress.

Here, we evaluated the drought tolerance of 52 *E. sibiricus* genotypes in seedlings. We identified drought tolerant (DT) and sensitive (DS) genotypes and compared the physiological, morphological, and ultrastructural responses of *E*. *sibiricus* with these genotypes under osmotic stress to elucidate the effects of root apoplastic barriers on drought tolerance. We also discuss whether differences in apoplastic barrier formation contribute to natural variations in drought tolerance between the two genotypes.

## Materials and methods

### Plant materials, growth conditions and treatments

Seeds from 52 wild *E*. *sibiricus* genotypes collected at the Sichuan Zoige Alpine Wetland Ecosystem National Observation and Research Station (**Supplementary Table 1**) were vernalized for 3 days in the dark at 4°C, then germinated on wet filter paper. The germinated seedlings were transferred to pots containing peat soil (Pindstrup, Mosebrug, Denmark) and watered compound fertilizer (N: P: K = 1:1:1) every 2 days. Ten-day-old seedlings were continuously watered or not (control) with distilled water for 8 days to evaluate the drought tolerance of germplasm resources. Seedlings were also transferred to aerated plastic boxes containing half‐strength Hoagland solution (H353, Phyto TechLabs, Lenexa, KS, USA) for hydroculture. Thereafter, 28-day-old plants were placed in 0 (control) and 20% PEG 6000 (BioFroxx, Einhausen, Germany) with an osmotic potential of −1.38 MPa for 3 days, osmolality was determined using an OSMOMAT 3000-D osmometer (Gonotec GmbH., Berlin, Germany). Plants grown in PEG 6000 for 0, 3 and 24 h were assessed by real-time quantitative polymerase chain reaction (qRT-PCR). All plants were cultivated in a climatic chamber at day/night temperatures of 23°C/19°C under a light cycle of 16 h/8 h (light/dark), light intensity of 250 μmol m^−2^· s^−1^, and relative humidity of ~ 60%.

### Physiological evaluation of drought tolerance

Leaves were washed with distilled water and gently wiped. Chlorophyll fluorescence indexes including the initial fluorescence (Fo), maximum fluorescence under light (Fm’), maximum fluorescence under dark adaptation (Fm), difference between maximum fluorescence under light and minimum fluorescence (Fv’), difference between maximum fluorescence under dark adaptation and initial fluorescence (Fv), maximum photochemical efficiency of photosystem II in the dark-adapted state (Fv/Fm), electron transport rate (ETR), photochemical quenching coefficient (qP), and non-photochemical quenching coefficient (NPQ) were measured as described (Bhusal et al., 2021) using an Li-6800 portable photosynthetic instrument (LI-COR Biosciences Inc., Lincoln, NE USA). Chlorophyll contents were measured in alcohol and acetone as described by Zhang et al. (2022) using a CARY60 UV spectrophotometer (Agilent Technologies Inc., Santa Clara, CA, USA). Relative electric conductivity (REC; a.k.a. electrolyte leakage) was measured as described by Wang et al. (2020a) and membrane lipid peroxidation was assessed as malondialdehyde (MDA) contents using the thiobarbituric acid reaction as described by Niu et al. (2016).

### Measurement of root hydraulic conductivity

Plants grown in 20% PEG 6000 were transferred back to half-strength Hoagland nutrient solution at least 1 h before measurements, based upon which hydrostatic hydraulic conductivity (*Lp*_r_) was calculated as *Lp*_hy_. The osmotic *Lp*_r_ (*Lp*_os_) was measured by replacing the nutrient solution with 1/2 Hoagland solution containing 30 mM NaCl at least 1 h before measurements. Five independent biological replicates per experiment were evaluated.

The *Lp*_r_ (including *Lp*_hy_ and *Lp*_os_) was measured using a high conductance flow meter (HFCM; Dynamax Inc., Houston, TX, USA) to determine the hydraulic conductance of the whole-plant water transport pathway. Samples were cut 4 cm above the basal root, and stumps were immediately connected to the HCFM that perfuses degassed water throughout the root system by applying pressure to a water-filled bladder within the unit. The flow rate of water through the root was determined using the HCFM in transient mode, with flow measured under increasing pressure delivered by a nitrogen gas cylinder. The applied pressure was gradually increased from 6 to ~ 500 kPa over the course of ~ 1 min, and the flow rate was logged every 2 s using Dynamax software. A transient curve was constructed, then the *Lp*_r_ was calculated as:


$$Lp_{r}=\;Qv/P$$

where Qv is the volumetric flow rate (kg·s^−1^) and P is the applied pressure (MPa). The temperature was automatically recorded by the HCFM, and all conductance measurements were corrected to values at 25°C. Hydraulic conductance was calculated using a transient increase in pressure with simultaneous recording of volume flow and normalized by dividing conductance by the total surface areas of the root (Tsuda and Tyree, 2000; Rodríguez-Gamir et al., 2019).

### Trisodium-8-hydroxy-1,3,6-pyrenetrisulphonic acid (PTS) analyses

Apoplastic bypass flow was analyzed using the water-soluble, fluorescent, and nontoxic tracer trisodium-8-hydroxy-1,3,6-pyrenetrisulphonic acid (PTS; Sigma-Aldrich Corp., St. Louis, MO, USA) that does not cross cell membranes or adhere to cell walls (Faiyue et al., 2010b; Krishnamurthy et al., 2011). Four-week-old plants were placed in 20% PEG 6000, 0.2 mM PTS (100 mg·L^−1^), and 0.2 mM PTS plus 20% PEG 6000 for 72 h, or normal half-strength Hoagland nutrient solution (control). Shoots were harvested and dried in an oven at 80°C for 72 h. Dry samples were immersed in 10 mL of distilled water for 2 h at 90°C. PTS florescence was analyzed at Λ_ex_ = 380 nm and Λ_em_ = 510 nm using a Varioskan LUX microplate reader (Thermo Fisher Scientific Inc., Waltham, MA, USA).

### Elemental analyses of shoots

Plants were removed from the osmotic environment, washed with distilled water to remove surface salts. Then the shoots were harvested and dried at 80°C for 72 h. Ground dried samples (~ 0.5 g) were mixed with the internal standard indium and digested in a muffle furnace with 5 mL of concentrated nitric acid at 170°C for 4 h. The digest was cooled to room temperature and the acid was evaporated almost to dryness then diluted to a final volume of 25 mL with 18 MΩ water to extract ions. The contents of B, Na, Mg, K, Al, Ca, Mn, Fe, Ni, Cu and Zn were determined using a Thermo X series II, inductively coupled plasma mass spectrometer (ICP-MS; Thermo Fisher Scientific Inc.), as described by the manufacturer.

### Root morphology and architecture

The roots of treated hydroponic plants were analyzed using a 12000XL scanner (Seiko Epson Corp., Tokyo, Japan), then images were analyzed using WinRHIZO2017 software (Regent Instruments, Sainte Foy, PQ, Canada) to obtain root parameters, namely total length, seminal root length and average diameter, surface area, forks, and fractal dimensions.

### Histochemical detection of CS and SL

Seminal root materials were fixed in FAA (50% ethanol, 5% glacial acetic acid and 5% formaldehyde) and dehydrated in a graded ethanol series. Cross-sections were cut at 12%, 24%, 36%, 48%, and 60% of the total seminal root length from the tip, along the entire seminal root, using a Cryotome-H-E cryostat microtome (Thermo Fisher), to detect development of the CS and SL over the root length. Development of the CS was detected by staining with 0.1% (w/v) berberine hemisulfate for 1 h and with 0.5% (w/v) aniline blue for 0.5 h (Brundrett et al., 1988). The SL was stained with 0.01% (w/v) lipophilic Fluorol Yellow 088 (FY088; Sigma-Aldrich Corp.) for 0.5 h and with 0.5% (w/v) aniline blue for 0.5 h (slightly modified from Lux et al., 2005). Stained cross‐sections were visualized using a DS-U3 epifluorescence microscope (Nikon Corp., Tokyo, Japan) with an ultraviolet filter set (excitation filter 361–389 nm, dichroic mirror 415 nm, barrier filter 430–490 nm) and photographed using a Nikon Eclipse camera at ISO 200 or 400 and 1–2 s exposure (Kreszies et al., 2018).

### Chemical analysis of suberin in roots

Root tissues (~500 mg fresh weight per sample) were rinsed with deionized water and dried on paper towels. Suberin cannot be directly quantified due to being insoluble and having a complex structure. Samples were delipidated and depolymerized to release monomers, then derivatized using N,O Bis-trimethylsilyl-trifluoroacetamide as described by Jenkin and Molina (2015).

Suberin monomers were identified and quantified using an 8890-7000D gas chromatograph-mass spectrometer (GC-MS; Agilent Technologies) fitted with an HP-5MS capillary column (length, 30 m; i.d., 0.25 mm; film thickness, 0.25 μm). The injector was set at 250°C, the injected split ratio was 1:10 and helium was the carrier gas at a constant flow of 1.0 mL min^−1^. The oven was initially set at 80°C for 2 min, increased by 15°C min^−1^ increments to 260°C, held for 10 min, then increased by 5°C min^−1^ increments to 320°C, and held for 24 min (total run time, 60 min). The temperature of the MS detector was 325°C, and the MS was set to a scan mode > 40–600 amu (electron impact ionization). Four biological replicates per experiment were assessed.

### qRT-PCR analysis

Total RNA was isolated using RNAiso Plus Mini Kits (Takara Bio Inc., Kusatsu, Japan). Complementary DNA was synthesized using RT OR-Easy TM II cDNA synthesis kits (Foregene, Chengdu, China). **Supplementary Table 2** lists the primers that were designed using Primer Premier 6.0. Primer specificity was validated based on melting profiles. The qRT-PCR proceeded using a StepOne Plus RT-PCR System (Thermo Fisher Scientific Inc.) and SYBR Premix Ex Taq (Toyobo, Tokyo, Japan). Relative expression was normalized to that of the housekeeping gene DnaJ (encodes heat shock N-terminal domain-containing protein) based on the 2^−ΔΔCt^ method because this is the most stable internal gene in *E. sibiricus* under osmotic stress in qRT-PCR analyses (Zhang et al., 2019). All experiments included three technical and three biological replicates.

### Statistical analyses

All results for traits in the evaluation of drought tolerance were converted into relative values to reduce inherent differences among different germplasm resources as:


trait relative value = Xs/Xc

where Xs and Xc represent drought stress and control, respectively (Zhang et al., 2022). The coefficient of variation for drought (CV_D_) was calculated as:


$$CV_{D}=|CV_{T}-CV_{C}|/\left(CV_{T}+CV_{C}\right)/2$$

where CV_T_ and CV_C_ respectively represent the coefficients of variation (CV) of all tested materials under drought stress and control conditions. Principal component analysis was carried out using SPSS 20.0 (IBM Corp., Armonk, NY, USA). Correlations among traits were determined using Pearson correlation coefficients (Adler and Parmryd, 2010). Drought resistance (D) values for the drought tolerance capacity of each genotype were calculated using subordinate function analysis as described by Yan et al. (2020).

All other data were statistically analyzed by one-way analysis of variance (ANOVA) using SPSS 20.0. Values are shown as means ± standard deviation (SD). Significant differences between means were determined using Duncan multiple range tests. Values with P< 0.05 were considered statistically significant. Histograms and linear graphs were generated using Origin 2019 (OriginLab Corp., Northampton, MA, USA).

## Results

### Comprehensive evaluation of drought tolerance of *E. sibiricus* genotypes

Eight days after drought stress, 13 physiological traits associated with photosynthetic pigment contents, relative plasma membrane permeability, and chlorophyll fluorescence parameters were comprehensively assessed in the 52 *E. sibiricus* genotypes. These traits changed to varying degrees among the genotypes, and the CV_D_ of REC and Fv/Fm were > 1 (100%). This indicated that these two traits were the most representative and were highly sensitive in the drought tolerance evaluation. Except for Fo and Fm, the CV_D_ of the other 11 traits were all > 0.10 (10%) (**Supplementary Table 3**).

We then calculated the variance contribution of 11 traits (excluding Fo and Fm) using principal component analysis (PCA). The Eigen values of the top two principal components were > 1, and the cumulative variance contribution rate was 66.04% (**Table 1**). The Eigen value of the first principal component was 5.63 with a variance contribution rate of 51.18% (**Table 1**). Chlorophyll fluorescence parameters, including ΦPS II, NPQ, qP, Fv’/Fm’, and Fv/Fm, as well as MDA, had the top six highest factor load capacities (**Table 1**). These six traits closely correlated (*P*< 0.01); MDA correlated positively with NPQ and negatively with the other four traits (**Supplementary Figure 1**). Chl a and Chl b had the top two highest factor load capacities in the second principal component (**Table 1**).

**Table 1:** Variance contribution of the top five principal components and factor load capacity.

Traits	Principal component				
	1	2	3	4	5
**Chl a**	0.542	0.726	-0.126	0.183	-0.125
**Chl b**	0.465	0.762	0.038	0.304	-0.068
**Car**	0.666	0.364	-0.157	-0.466	0.125
**REC**	-0.344	0.295	0.845	-0.100	0.203
**MDA**	-0.845	0.026	0.113	0.265	-0.058
**Fv/Fm**	0.743	-0.368	0.080	0.216	-0.303
**NPQ**	-0.846	0.294	-0.165	0.030	0.058
**ETR**	0.612	-0.175	-0.109	0.323	0.684
**qP**	0.832	-0.117	0.324	-0.111	-0.111
**ΦPSII**	0.911	0.024	-0.012	-0.222	0.053
**Fv’/Fm’**	0.824	-0.197	0.182	0.268	-0.076
**Eigen value**	5.630	1.634	0.953	0.708	0.664
**Variance contribution (%)**	51.178	14.859	8.663	6.438	6.039
**Cumulative contribution (%)**	51.178	66.037	74.700	81.138	87.177

The data of the top five principal components were calculated based on the data shown in **Supplementary Table 2**. Car, carotenoid; Chl a, chlorophyll a; Chl b, chlorophyll b; ETR, electron transport rate, Fv/Fm, PS II maximum photochemical quantum yield; Fv’/Fm’, PSII effective photochemistry quanta output; MDA, malondialdehyde content; NPC, non-photochemical quenching coefficient; qP, photochemical quenching coefficient; REC, relative conductivity rate; ΦPSII, quantum yield of PSII electron transport.

The relative drought tolerance reflected by the D values of the other 11 traits for each genotype were calculated according to the trait relative value (**Supplementary Table 4**), and ranked by subordinate function (**Table 2**). Three genotypes, B-12-9-1, I-1-5-46, and I-1-5-63, with D values > 0.95, were the most drought tolerant genotypes. The other three genotypes, I-1-5-2, I-1-5-3, and I-1-5-53, with D values< 0.20, were the most drought-sensitive genotypes (**Table 1**). We then selected I-1-5-46 and I-1-5-2 as the DT and DS genotypes for further analysis.

**Table 2:** Comprehensive evaluation through subordinate functions of 52 *Elymus sibiricus* genotypes.

Subordinate function value													
ID	Chl a	Chl b	Car	REC	MDA	Fv/Fm	NPQ	ETR	qP	ΦPSⅡ	Fv’/Fm’	D Values	Rank
**B-12-9-1**	0.93	0.91	1.00	0.98	1.00	0.93	0.99	0.98	0.99	0.98	0.95	0.97	1
**I-1-5-46**	0.96	0.81	0.96	0.98	1.00	0.96	0.99	0.96	1.00	1.00	0.93	0.96	2
**I-1-5-63**	0.93	0.91	0.96	0.99	0.97	0.90	1.00	0.95	0.99	0.97	0.96	0.96	3
**14-091**	0.90	0.82	0.75	0.93	0.61	0.67	0.94	0.59	0.76	0.71	1.00	0.79	4
**10-5**	0.90	0.96	0.79	0.80	0.66	0.68	0.91	0.35	0.87	0.63	0.69	0.75	5
**09-152**	0.75	0.29	0.87	0.98	0.68	0.76	0.95	0.62	0.73	0.67	0.90	0.75	6
**I-1-5-60**	1.00	1.00	0.36	0.98	0.65	0.61	0.94	0.60	0.72	0.57	0.62	0.73	7
**I-1-5-39**	0.55	0.54	0.96	0.91	0.68	0.68	0.94	0.76	0.81	0.57	0.63	0.73	8
**09-280**	0.79	0.95	0.89	0.57	0.62	0.88	0.95	0.40	0.75	0.57	0.58	0.72	9
**I-1-5-49**	0.98	0.58	0.53	0.89	0.69	0.81	0.94	0.50	0.60	0.53	0.89	0.72	10
**08-129**	0.65	0.57	0.70	0.86	0.48	0.84	0.89	0.79	0.83	0.55	0.70	0.71	11
**09-244**	0.26	0.67	0.98	0.79	0.65	0.81	0.86	0.34	0.87	0.64	0.86	0.70	12
**I-1-5-23**	0.87	0.85	0.84	0.70	0.45	0.68	0.94	0.54	0.52	0.54	0.79	0.70	13
**I-1-5-25**	0.72	0.86	0.48	0.72	0.51	0.85	0.94	0.50	0.95	0.43	0.74	0.70	14
**09-183**	0.76	0.44	0.80	0.99	0.68	0.81	0.93	0.21	0.76	0.59	0.68	0.70	15
**I-1-5-66**	0.77	0.42	0.95	1.00	0.85	0.61	0.76	0.33	0.63	0.63	0.61	0.69	16
**I-1-5-42**	0.94	0.79	0.59	0.65	0.68	0.69	0.93	0.48	0.64	0.55	0.48	0.67	17
**I-5-14**	0.45	0.71	0.49	0.97	0.66	0.66	0.93	0.68	0.67	0.57	0.61	0.67	18
**SWUN 2**	0.54	0.49	0.97	0.48	0.82	0.68	0.88	0.80	0.61	0.55	0.52	0.67	19
**09-055**	0.20	0.26	0.71	0.93	0.62	0.85	0.94	0.62	0.66	0.64	0.89	0.67	20
**I-1-5-45**	0.61	1.00	0.53	0.24	0.67	0.68	0.86	0.68	0.68	0.55	0.76	0.66	21
**I-1-5-61**	0.43	0.86	0.35	0.52	0.65	0.73	0.77	0.83	0.68	0.55	0.87	0.66	22
**11-14**	0.52	0.30	0.85	0.62	0.57	0.61	0.94	0.88	0.69	0.64	0.58	0.65	23
**09-089**	0.94	0.91	0.84	0.51	0.60	0.13	0.65	0.40	0.83	0.63	0.62	0.64	24
**I-1-5-67**	0.32	0.37	0.56	0.82	0.68	0.57	0.94	0.70	0.84	0.62	0.64	0.64	25
**I-1-4-12**	0.32	0.65	0.51	0.77	0.53	1.00	0.94	0.17	0.87	0.51	0.78	0.64	26
**14-16-2**	0.39	0.32	0.95	0.87	0.62	0.67	0.92	0.37	0.70	0.62	0.60	0.64	27
**I-1-5-50**	0.29	0.23	0.54	0.92	0.69	0.75	0.85	0.64	0.80	0.62	0.70	0.64	28
**I-5-2-9**	0.91	0.64	0.49	0.51	0.60	0.61	0.72	0.49	0.68	0.58	0.78	0.64	29
**I-1-5-30**	0.63	0.42	0.52	0.88	0.67	0.66	0.87	0.65	0.64	0.51	0.55	0.64	30
**I-1-5-21**	0.07	0.50	0.68	0.84	0.45	0.62	0.95	1.00	0.61	0.45	0.78	0.63	31
**I-1-4-1**	0.60	0.62	0.35	0.63	0.48	0.75	0.93	0.68	0.61	0.48	0.80	0.63	32
**09-149**	0.14	0.22	0.69	0.91	0.64	0.35	0.92	0.79	0.76	0.61	0.65	0.61	33
**09-083**	0.55	0.35	0.61	0.37	0.61	0.65	0.94	0.34	0.83	0.59	0.83	0.61	34
**09-124**	0.48	0.78	0.57	0.30	0.66	0.33	0.93	0.35	0.83	0.72	0.61	0.60	35
**I-1-5-18**	0.21	0.25	0.57	0.80	0.62	0.68	0.88	0.43	0.74	0.64	0.73	0.60	36
**I-1-5-29**	0.25	0.33	0.22	0.90	0.56	0.78	0.87	0.69	0.61	0.52	0.69	0.58	37
**I-1-5-71**	0.04	0.45	0.56	0.53	0.85	0.56	0.92	0.26	0.86	0.58	0.65	0.57	38
**14-694**	0.56	0.55	0.74	0.00	0.44	0.56	0.89	0.31	0.95	0.69	0.57	0.57	39
**I-1-5-41**	0.04	0.00	0.20	0.65	0.54	0.79	0.92	0.73	0.90	0.49	0.95	0.56	40
**I-1-5-20**	0.11	0.20	0.37	0.69	0.64	0.87	0.94	0.32	0.69	0.59	0.78	0.56	41
**I-1-5-59**	0.11	0.03	0.19	0.97	0.69	0.72	0.95	0.53	0.77	0.52	0.70	0.56	42
**I-1-3-3**	0.29	0.15	0.34	0.27	0.57	0.85	0.94	0.73	0.86	0.40	0.74	0.56	43
**B-12-13-2**	0.22	0.38	0.66	0.76	0.38	0.78	0.85	0.51	0.68	0.41	0.49	0.56	44
**I-1-5-28**	0.58	0.35	0.44	0.41	0.49	0.74	0.94	0.26	0.70	0.50	0.69	0.55	45
**I-1-5-40**	0.15	0.12	0.78	0.37	0.67	0.69	0.95	0.52	0.65	0.55	0.56	0.55	46
**I-1-6-2**	0.09	0.30	0.11	0.62	0.47	0.66	0.95	0.80	0.69	0.59	0.72	0.55	47
**09-071**	0.09	0.21	0.08	0.98	0.55	0.81	0.86	0.21	0.84	0.55	0.75	0.54	48
**I-1-5-58**	0.00	0.04	0.43	1.00	0.69	0.69	0.94	0.37	0.53	0.58	0.36	0.51	49
**I-1-5-53**	0.21	0.31	0.13	0.37	0.28	0.16	0.00	0.13	0.20	0.00	0.25	0.19	50
**I-1-5-3**	0.08	0.22	0.09	0.64	0.07	0.00	0.16	0.06	0.19	0.04	0.00	0.14	51
**I-1-5-2**	0.06	0.19	0.00	0.60	0.00	0.07	0.19	0.00	0.00	0.12	0.03	0.11	52

Car, carotenoid; Chl a, chlorophyll a; Chl b, chlorophyll b; D values, drought resistance values; ETR, electron transport rate; Fm, maximal fluorescence; Fo, minimal fluorescence; Fv/Fm, PS II, maximum photochemical quantum yield; Fv’/Fm’, PSII effective photochemistry quanta output; MDA, malondialdehyde content; NPC, non-photochemical quenching coefficient; qP, photochemical quenching coefficient; REC, relative conductivity rate; ΦPSII, quantum yield of PSII electron transport.

### Radial transport of water and nutrients in roots during water deficits

According to the composite transport model, radial water transport in plant roots can occur *via* apoplastic, symplastic and transcellular pathways (Kim et al., 2018; Kreszies et al., 2020). The radial water flow in plant roots is usually measured as hydraulic conductivity (*Lp*_r_ in m s^−1^ MPa^−1^). The hydrostatic *Lp*_r_ (*Lp_hy_*) determines the water flow through both the apoplastic and cell-to-cell pathways, and the osmotic *Lp*_r_ (*Lp_os_*) represents the water transport across the cell-to-cell pathway (Steudle, 2000; Kreszies et al., 2018). The ratios of *Lp*_hy_ to *Lp*_os_ indicate which pathway contributes more to the overall water transport across the root (Steudle and Peterson, 1998; Kreszies et al., 2018). Here, *Lp*_hy_ was higher in DS than in DT, whereas the trend of *Lp*_os_ was the opposite under non-stress conditions. Under osmotic stress, *Lp*_hy_ did not significantly differ between DS and DT, *Lp*_os_ was higher in DS than in DT, and *Lp*_os_ increased in DS but *Lp*_hy_ decreased in DT (**Table 3**). The ratio of *Lp*_hy_ to *Lp*_os_ was higher in control DS, but osmotic stress significantly decreased it to< 1 (**Table 3**).

**Table 3:** Hydrostatic and osmotic hydraulic conductivity (*Lp_r_*) in drought-tolerant (DT) and drought-sensitive (DS) genotypes grown under control or osmotic stress conditions.

Parameters	DS		DT	
	control	20% PEG	control	20% PEG
**Hydrostatic *Lp*_r_ (*Lp*_hy_) (10^-8^ m·s^-1^·Mpa^-1^)**	3.39 ± 1.60a	1.75 ± 0.5ab	1.56 ± 0.34b	0.92 ± 0.53b
**Osmotic *Lp*_r_ (*Lp*_os_) (10^-8^ m·s^-1^·Mpa^-1^)**	1.89 ± 0.51b	2.88 ± 0.53a	2.96 ± 0.27a	1.70 ± 0.69b
**Hydrostatic/Osmotic (*Lp*_hy_/*Lp*_os_)**	1.89 ± 0.50a	0.62 ± 0.13b	0.53 ± 0.05b	0.61 ± 0.24b

Mean values of *Lp*_hy_ and *Lp*_os_ were calculated for the total root systems of individual plants. Results are given as mean with standard deviation (SD) of five independent replicates (n = 5). Different letters indicate significant differences at *P <* 0.05 based on one‐way analysis of variance (ANOVA) (Fisher’s Duncan test).

**Figure 1:**
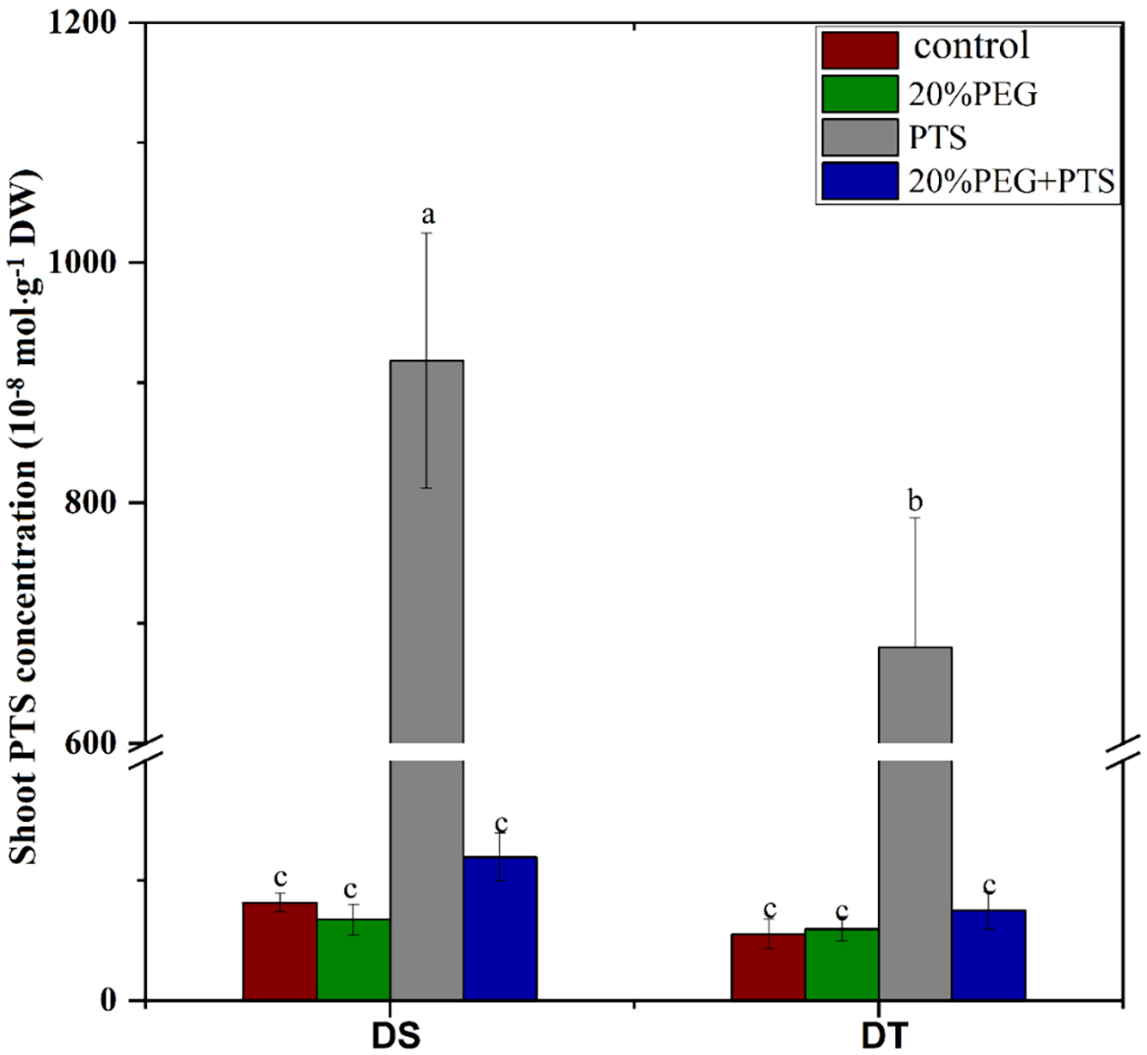
Trisodium-8-hydroxy-1,3,6-pyrenetrisulfonic acid (PTS) concentration in shoots of drought-tolerant (DT) and drought-sensitive (DS) genotypes grown under control or osmotic stress conditions. Plants were treated with 0.2 mM PTS or 0.2 mM PTS plus 20% (w/v) PEG 6000 for 72 h. Treatment without PTS was used as the negative control. Values are the mean ± standard deviation (SD) (n = 5). Different letters indicate significant differences at P < 0.05 based on one-way analysis of variance (ANOVA) (Fisher’s Duncan test). DW, dry weight.

The fluorescent dye, PTS, is a tracer for the apoplastic pathway translocation of solutes as it does not cross cell membranes or adhere to cell walls (Yeo et al., 1987; Faiyue et al., 2010b; Wang et al., 2020a). Therefore, we investigated the apoplastic bypass flow of solutes in *E. sibiricus* using this dye. In the absence of osmotic stress, the shoot PTS concentration was significantly higher in DS than in DT, indicating that DS possessed more apoplastic pathway radial transport of solutes than DT, which was consistent with the apoplastic transport of water (**Table 3**). Osmotic stress abolished apoplastic solute influx in both DT and DS (**Figure 1**).

**Figure 2:**
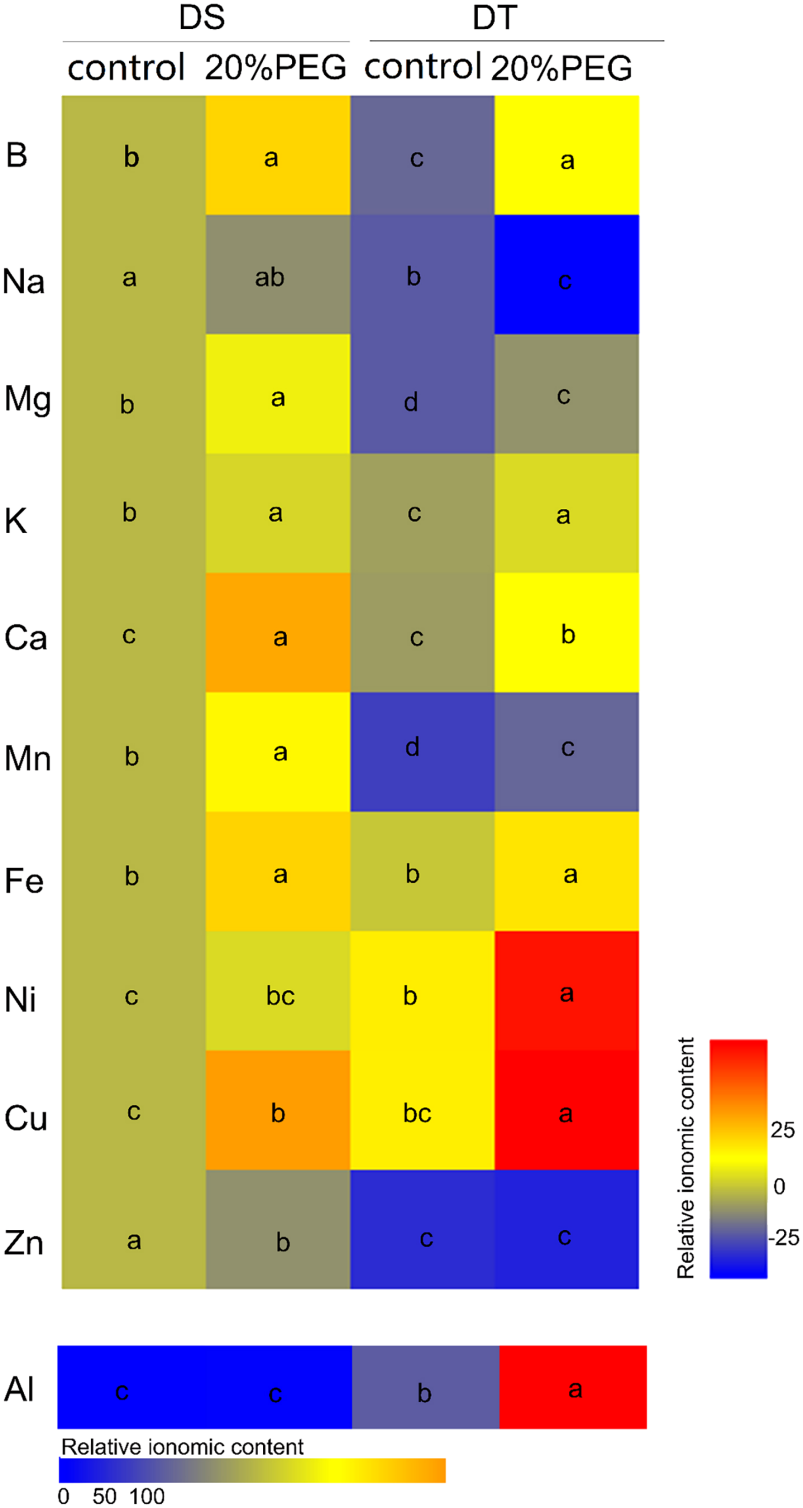
Mineral elemental accumulation in shoots of drought-tolerant (DT) and drought-sensitive (DS) genotypes grown under control or osmotic stress conditions. Mineral elemental concentrations were determined using inductively coupled plasma mass spectrometry (ICP-MS). Results are presented relative to the control DS values calculated from numeric values presented in **Supplementary Table 5**. Different letters indicate significant differences at *P <* 0.05 based on one-way analysis of variance (ANOVA) (Fisher’s Duncan test).

We compared the mineral element profiles in shoots between DS and DT using ICP-MS. The concentrations of B, Na, Mg, K, Ca, Mn and Zn were significantly higher in DS than in DT under either control or osmotic stress conditions. However, the concentrations of Ni, Cu, and Al were lower in DS than in DT (**Figure 2** **;** **Supplementary Table 5**). Osmotic stress induced the accumulation of B, Mg, K, Ca, Mn, Fe, and Cu in shoots of DT and DS but increased the concentration of Ni and Al only in DT. Osmotic stress reduced Na accumulation in DT and Zn accumulation in DS (**Figure 2** **;** **Supplementary Table 5**).

### Root morphology and anatomy

**Figure 3:**
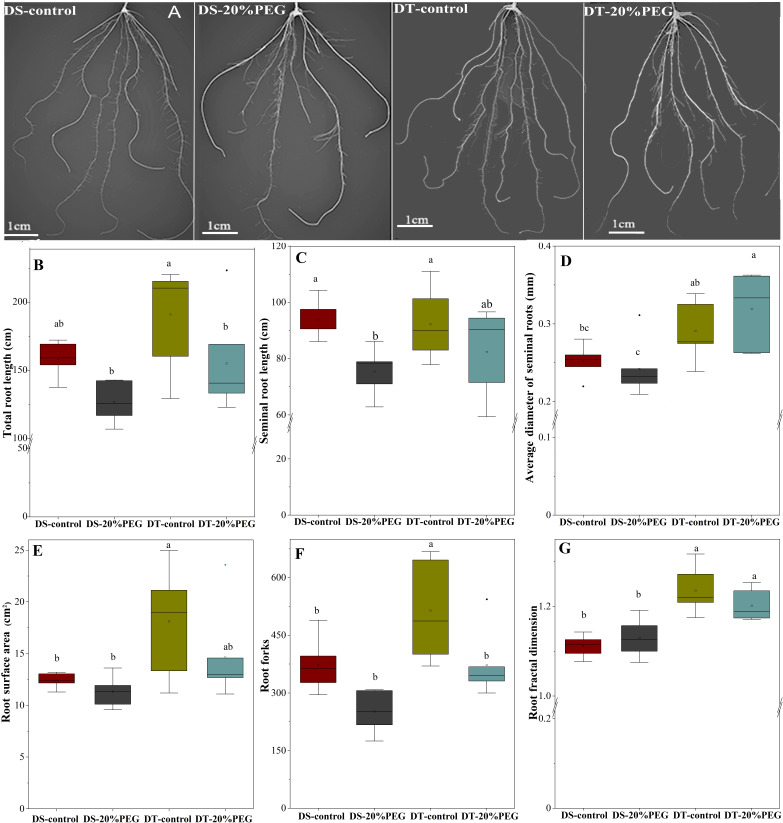
Root morphology and architecture of drought-tolerant (DT) and drought-sensitive (DS) genotypes grown under control or osmotic stress conditions. **(A)** Root images. **(B)** Total root length. **(C)** Seminal root length. **(D)** Average diameter of seminal roots. **(E)** Root surface area. **(F)** Root forks. **(G)** Root fractal dimension. All values represent the roots of each plant and are shown as mean with standard deviation (SD) of five independent replicates (n = 5). Different letters indicate significant differences at *P <* 0.05 based on one-way analysis of variance (ANOVA) (Fisher’s Duncan test).

We investigated the effects of PEG-induced osmotic stress on the root morphology and architecture of DT and DS to determine why root radial transport of water and mineral nutrients differed between the genotypes **(** **Figures 3A–G** **)**. The results showed that DT had a larger root surface area, more root forks, and a higher root fractal dimension than DS under control conditions **(** **Figures 3E–G** **)**. Osmotic stress reduced the seminal root length in DS but not in DT **(** **Figure 3C** **)**, and decreased total root length and root forks in DT, but not in DS **(** **Figure 3A, F** **)**. The average diameter of seminal roots and the root fractal dimension were significantly higher in DT than in DS under osmotic stress **(** **Figures 3D, G** **)**.

We assessed the development of CS and SL in roots by histochemical staining. Both the CS and SL were obvious in the endodermis, but not in the hypodermis, even at 60% of the total root length from the tip, under osmotic stress either in DT or DS (**Supplementary Figure 2**). Therefore, when grown hydroponically, seminal roots of *E. sibiricus* did not develop an exodermis, even under osmotic stress.

**Figure 4:**
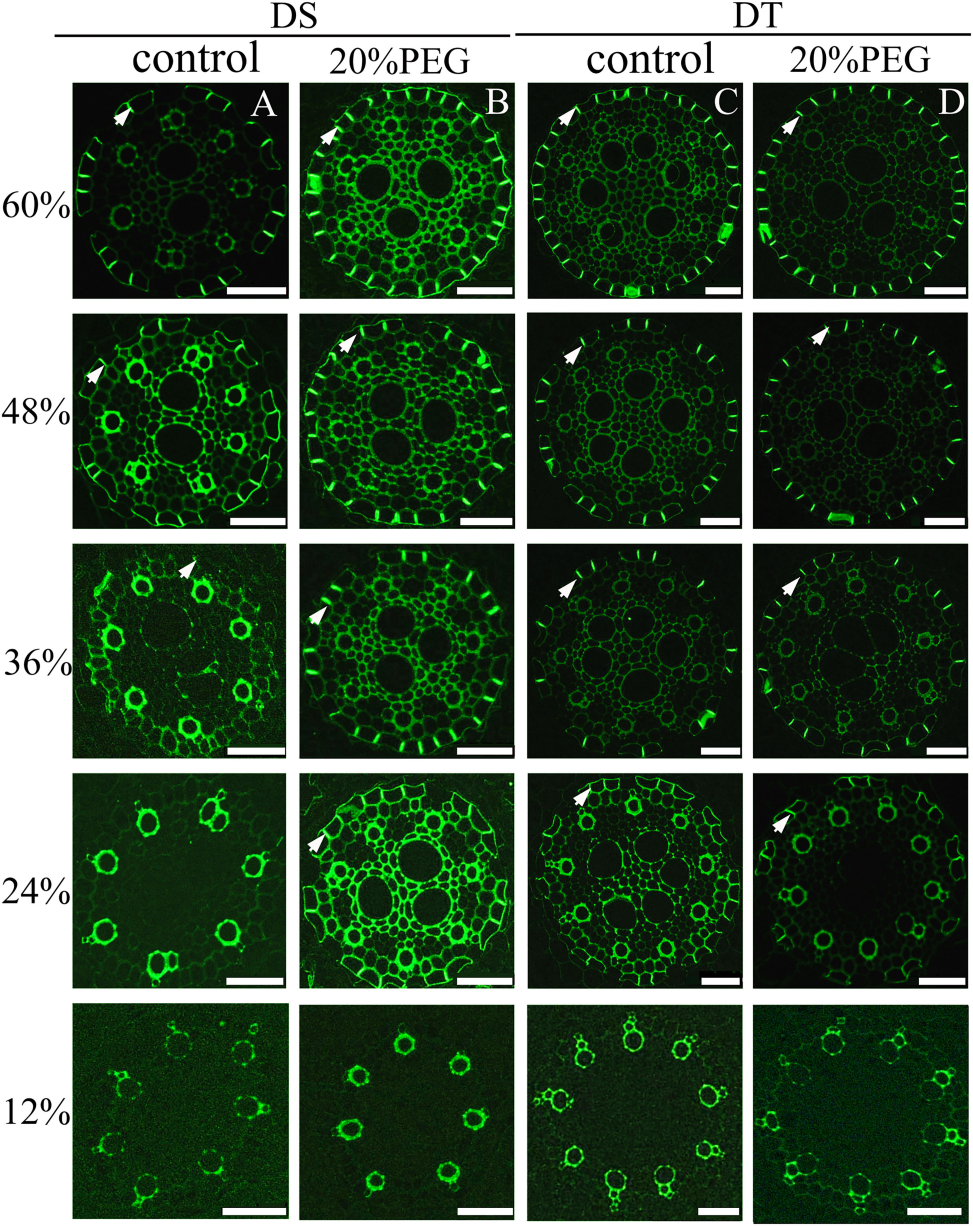
Fluorescence staining of Casparian strip (CS) in seminal roots of drought-tolerant (DT) and drought-sensitive (DS) genotypes grown under control or osmotic stress conditions. CS in seminal roots were stained with berberine hemisulfate for control DS **(A)**, osmotic-stressed DS **(B)**, control DT **(C)**, and osmotic-stressed DT **(D)**. Green fluorescent spots (white arrow) indicate the CS. Numbers on the vertical axis represent the distance from the tip as a percentage of the total root length (scale bars, 42.5 µm).

No CS was found at 12% of the root length in control and osmotic-stressed plants of both genotypes (**Figure 4**) and at 24% of the root length in the control DS. The first appearance of a weak “dot-like” CS signal in the control DS was found at 36% of the root length, and at 48%, a fully-formed CS appeared in many endodermal cells in the control DS (**Figure 4A**). Of note, a fully-formed CS continuously lines the entire radial wall of an endodermal cell, rather than having an initial dot-like structure (Kreszies et al., 2019; Li et al., 2020). The DS did not develop a complete CS even at 60% of the root length under control conditions (**Figure 4A**). The osmotically stressed DS developed a complete CS from 24% of the root length (**Figure 4B**). A fully-formed CS appeared in some endodermal cells at 24% of the root length in DT, and the CS was well-developed at 60% of the root length, with no obvious difference in DT between control and water-deficient conditions (**Figures 4C, D**).

**Figure 5:**
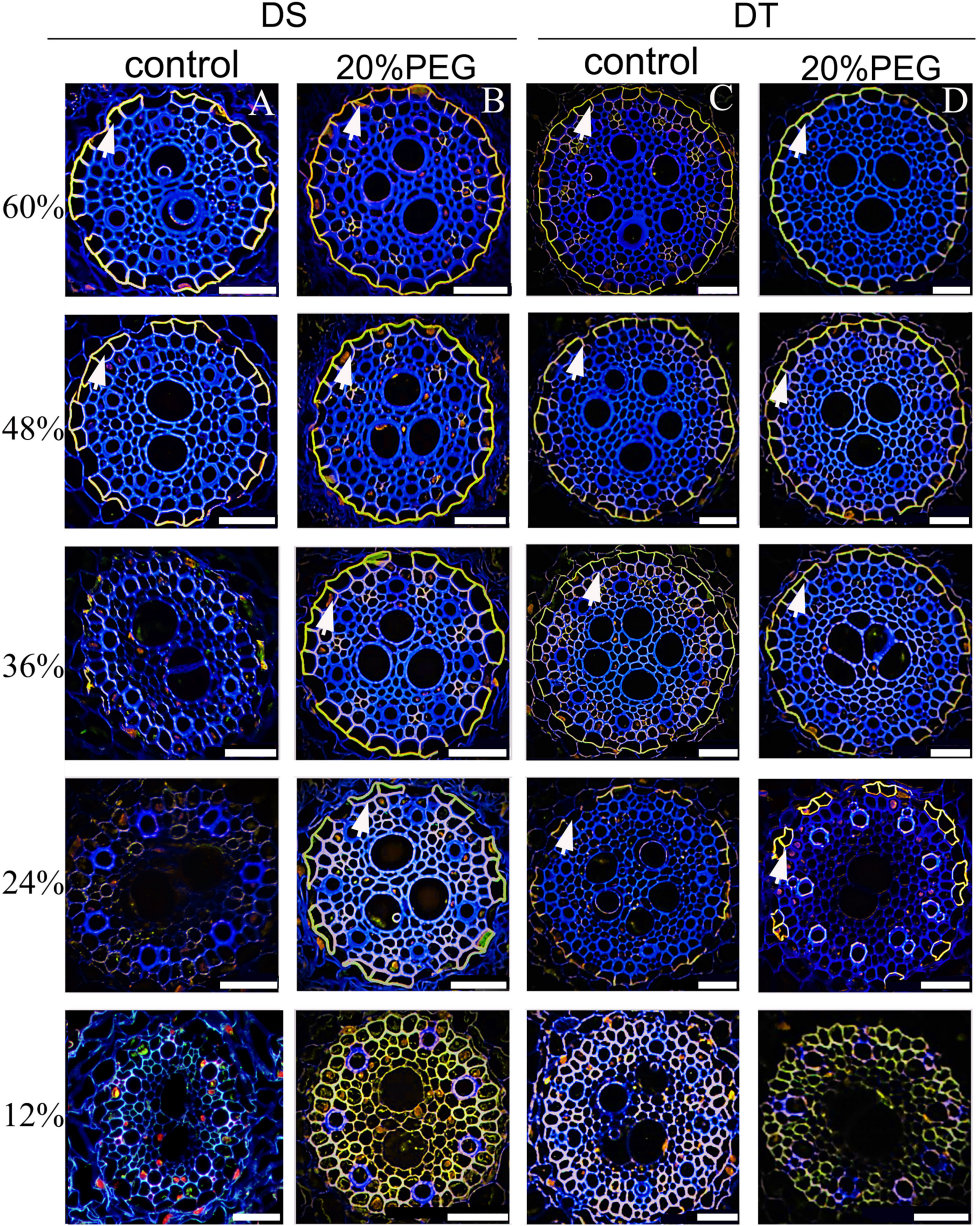
Fluorescence staining of suberin lamellae (SL) in seminal roots of drought-tolerant (DT) and drought-sensitive (DS) genotypes grown under control or osmotic stress conditions. The deposition of the SL in seminal roots was visualized by staining with Fluorol Yellow 088 for control DS **(A)**, osmotic-stressed DS **(B)**, control DS **(C)**, and osmotic-stressed DT **(D)**. The yellow fluorescent rings (white arrow) indicate the SL. Numbers on the vertical axis represent the distance from the tip as a percentage of the total root length (scale bars, 42.5 µm).

The developmental trend of the SL was very similar (**Figure 5**). The SL was not detectable at 12% of the root length in all plants. Patchy development of the SL was evident at 48% and 60% of the root length in the control DS (**Figure 5A**). The osmotically stressed DS had a patchy SL at 24% and 36%, and a well-developed SL at 48% and 60% of the root length. Very few endodermal cells without an SL were taken as passage cells (**Figure 5B**). A patchy SL was visible from 24%–48% along the main axis of the roots in control DT and detectable at 24%–36% of the root length in osmotically stressed DT. The SL was fully deposited at 48% and 60% of the root length in DT plants treated with PEG and was completely formed in control DT at 60% (**Figures 5C, D**).

### Chemical analysis of suberin of *Elymus sibiricus* in response to osmotic stress

**Figure 6:**
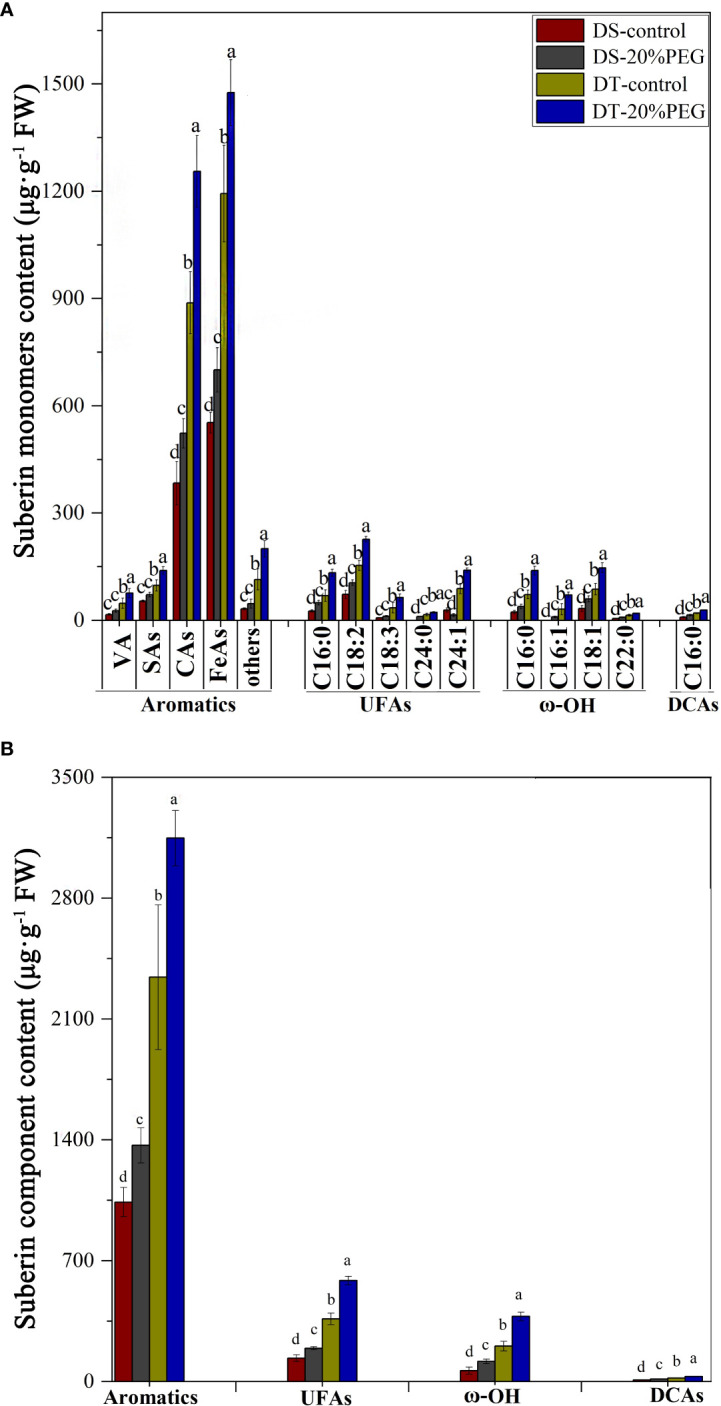
Amount of suberin in roots detected in drought-tolerant (DT) and drought-sensitive (DS) genotypes grown under control or osmotic stress conditions. **(A)** Amount of all detected monomers of suberin in roots. The substance classes of aromatic components include vanillin (VA), salicylic acids (SAs), coumaric acids (CAs), ferulic acids (FeAs), and others; the substance classes of aliphatic components included unsubstituted fatty acids (UFAs), ω-hydroxy acids (ω-OH), and α,ω-dicarboxylic acids (DCAs). **(B)** Amounts of major substance classes of suberin. Absolute amounts of suberin are shown as mean values in μg·g^−1^ fresh weight (FW) ± the standard deviation (SD) of four biological replicates (n = 4). Different letters indicate significant differences at *P <* 0.05 based on one-way analysis of variance (ANOVA) (Fisher’s Duncan test).

Suberin monomer contents were analyzed to further determine differences between DT and DS in terms of root apoplastic barriers under control and osmotic stress conditions. The monomer classes in *E. sibiricus* aliphatic suberin comprised unsubstituted fatty acids (UFAs), α,ω-dicarboxylic acids (DCAs), and ω-OH acids. The most abundant aliphatic suberin monomers were UFAs and ω-OH acids. The chain lengths of the aliphatic suberin monomers varied from C16 to C24. The aromatic suberin monomer in *E. sibiricus* root comprised a series of different substance classes, namely vanillin (VA), salicylic (SAs), coumaric (CAs), and ferulic (FeAs) acids, of which FeAs and CAs were the most abundant aromatic components (**Figure 6A**). Aromatic suberin accounted for 76%–83% of the total suberin content (**Figure 6A**), which is consistent with that in other Gramineae species, such as rice and barley (Kreszies et al., 2018).

The abundance of all suberin monomers was significantly higher in DT than in DS (**Figures 6A, B**). The total suberin content was ~ 135% and 144% higher in DT than that in DS under control and osmotic stress conditions, respectively. Osmotic stress increased the suberin contents in DS and DT plants by ~ 35% and 41%, respectively, compared with controls (**Figure 6C**). Nevertheless, osmotic stress induction did not compensate for the lower suberin content in DS compared with DT (**Figures 6A, B**). Osmotic stress induced significant amounts of suberin, which closely corresponded with the histochemical staining results for SL in DS, but not in DT (**Figure 5**).

### Expression of genes associated with apoplastic barriers

**Figure 7:**
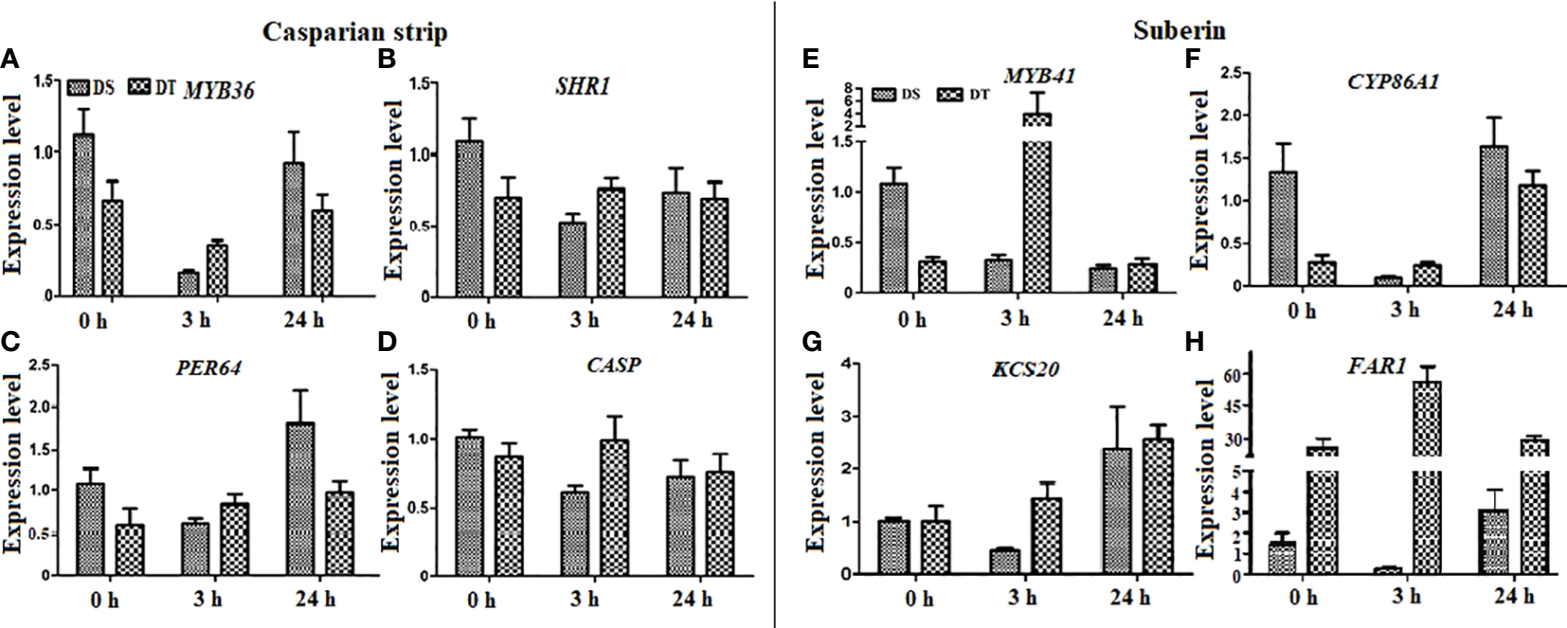
Expression levels of apoplastic barrier-related genes in drought-tolerant (DT) and drought-sensitive (DS) genotypes under osmotic stress conditions.Twenty-eight-day-old seedlings were subjected to 20% (w/v) PEG 6000 treatment for 0, 3, and 24 h. **(A–D)** Expression levels of Casparian strip-related genes. **(E–H)** Expression levels of suberin-related genes. *DnaJ* was used as a reference gene, and the relative expression levels of all genes were assessed using qRT-PCR. Values are mean ± standard deviation (SD) (n = 3).

The relative expression of eight genes related to apoplastic barriers in roots were investigated by qRT-PCR. Among them, four each that were respectively associated with CS formation and suberin monomer synthesis comprised *MYB36*, *SHR1* (also regulates root suberization), *PER64*, and *CASP* (Naseer et al., 2012; Hosmani et al., 2013; Kamiya et al., 2015; Wang et al., 2020b; Wang et al., 2022a; Xu et al., 2022), and *MYB41*, *CYP86A1*, *KCS20*, and *FAR1* (Höfer et al., 2008; Lee et al., 2009; Domergue et al., 2010; Kosma et al., 2014; Shukla et al., 2021). Osmotic stress increased the expression of *PER64* in DS (24 h), downregulated that of *MYB36*, *SHR1*, and *CASP* to varying degrees in DS, and only slightly affected that of the four genes related to CS formation in DT (**Figures 7A–D**). Osmotic stress for 24 increased the expression of *KCS20* and *FAR1* and decreased that of *MYB41* in DS. Osmotic stress for 3 h downregulated transcription of the four genes associated with suberin synthesis in DS, whereas that for either 3 or 24 h induced their expression in DT (**Figures 7E–H**).

## Discussion

Roots absorb water from soil, sense water deficits in dry soil, and transduce signals during water deficits. Water flow in roots, which is usually measured as *Lp*_r_, varies according to growth conditions (Baxter et al., 2009; Wang et al., 2020a). Here, we selected drought-tolerant (I-1-5-46) and sensitive (I-1-5-2) *E. sibiricus* genotypes *via* a comprehensive evaluation and compared their differential root response mechanisms to drought. We found significantly lower overall water flow and lower apoplastic bypass flow of solutes, in DT, than in DS, in the absence of PEG 6000. Water and solutes were absorbed exclusively *via* the cell-to-cell pathway in response to osmotic stress (**Table 3**, **Figure 1**). The CS and SL are important barriers affecting apoplastic bypass flow of water and solutes in roots, and thus potentially play roles in abiotic stress tolerance, such as drought (Hose et al., 2001; Geldner, 2013; Wang et al., 2022a). In *Arabidopsis*, the *cyp86a1*/*horst* mutant, with a deficiency of aliphatic suberin, has increased *Lp*_hy_, *Lp*_os_, and *Lp*_hy_/*Lp*_os_, suggesting that aliphatic suberin plays a role in limiting water flow through the apoplastic and transcellular pathway (Ranathunge et al., 2011b). Most radial water uptake in barley occurs through weakly suberized younger roots (Ranathunge et al., 2017). Our histochemical, root hydraulic conductivity and PTS tracer findings precisely corresponded. The DT plants developed a CS and SL closer to the root tips and contained more abundant suberin monomer contents than DS in the absence of osmotic stress (**Figures 4** **–** **6**). The proportion of apoplastic bypass flow was higher in DS when endodermal barriers were not completely established. However, the deposition of barriers in the zone near the root tip largely blocked apoplastic water uptake, and the cell-to-cell pathway became the dominant transport route for root water uptake. A similar phenomenon has been found in barley (Kreszies et al., 2020). The *Lp_os_* notably increased in DS in response to osmotic stress, which might be a compensation strategy associated with enhanced aquaporin activity and might represent an adaptative mechanism of *E. sibiricus* to ensure sufficient water uptake under osmotic stress. Water uptake *via* the cell‐to‐cell pathway mainly depends on plasma membrane aquaporins (Grondin et al., 2016). The contribution of this pathway to water uptake can be reversibly regulated by rapidly modulating the activity of aquaporins in barley roots (Kaneko et al., 2015). The decrease in *Lp_os_* in osmotically stressed DT plants might be interpreted as thickened SL deposits blocking endodermal cell walls, thus reducing water uptake through transcellular pathways. Variations in suberin monomer arrangements and their microstructure in different cultivars might contribute to water movement in roots (Tao et al., 2017). More in-depth investigation into these aspects of cell physiology and ultrastructural observations are needed. Like water transport, the radial transport of solutes in roots under osmotic stress relied on the cell-to-cell pathway. This was compatible with previous findings that PEG reduces bypass flow in rice (Yeo et al., 1999; Faiyue, 2010a).

Suberized cell walls in the endodermis/exodermis of roots form transport barriers to water and solutes (Peterson and Cholewa, 1998). In fact, suberization of the endodermis/exodermis is characterized by CS and SL deposition. The endodermis is not distinguished from the cortex before differentiation, whereas the exodermis is formed *via* specialization of the hypodermis (Barberon et al., 2016; Doblas et al., 2017). We found here that *E. sibiricus* did not develop an exodermis under normal conditions and even under osmotic stress (**Supplementary Figure 2**). This is consistent with the findings in *Arabidopsis* and barley but differ from that in other gramineous plants, such as rice, maize, and sheepgrass (*Leymus chinensis*), which develop a strong exodermis in response to stress (Schreiber et al., 2005; Kreszies et al., 2018; Kreszies et al., 2019; Li et al., 2020). *Elymus sibiricus* is currently the only known species among forage grasses that does not develop an exodermis, which might make it a good model for investigating endodermal barriers without interference from the outer parts of roots. A fraction of seminal roots in one wild barley accession (*Hordeum vulgare* spp. *spontaneum*) is induced to form a lignified and suberized exodermis in response to osmotic stress (Kreszies et al., 2018). Moreover, the wetland barley species, *Hordeum marinum*, generally forms and reinforces an exodermis to prevent radial oxygen loss when grown under stagnant conditions (Kotula et al., 2017). Whether *E. sibiricus* could form an exodermis in response to other stresses, such as salt, waterlogging, or cold, remains unknown. However, these grass plants that can survive and grow in such harsh environments for long periods might have better adaptive potential than their cultivated relatives.

Osmotic stress enhances suberization but not lignification in barley (Kreszies et al., 2018). Chronic drought increases root suberin content but does not alter its lamellar structure in *Arabidopsis* (de Silva et al., 2021). The induction or strengthening of apoplastic barriers is also very pronounced when rice is exposed to salinity (Krishnamurthy et al., 2009) or stagnant deoxygenated conditions (Ranathunge et al., 2011a). Moreover, natural variations in salt tolerance (Krishnamurthy et al., 2009; Krishnamurthy et al., 2011) and Cd accumulation (Qi et al., 2020) between rice cultivars have been attributed to differences in root apoplastic barriers. Here, the responses of the CS and SL to osmotic stress differed between genotypes of *E. sibiricus*, which is the first such finding in forage grasses. The suberin contents were higher under non-stressed conditions in DT, than in DS induced by osmotic stress. We speculated that the development of a precocious endodermal barrier under non-stress conditions, to cope with possible adverse environmental factors, might be an important survival strategy of DT plants. We found that osmotic stress induced the CS and SL of DS to form closer to root tips, whereas their development in DT was not obviously changed by water deficits (**Figures 4**, **5**). However, osmotic stress indeed increased suberin contents in DT. Therefore, the reinforcement of suberization induced by osmotic stress in DT was likely manifested by an increase in the thickness of the SL. However, this notion requires further verification by transmission electron microscopy. Such thickening of the CS has also been identified in response to salt stress in several plant species (Karahara et al., 2004; Prathumyot and Ehara, 2010; Al Kharusi et al., 2019; Cui et al., 2021; Wang et al., 2022b). The establishment of apoplastic barriers in lateral roots provides another explanation for the gap between observations based on the SL in seminal roots and the total root suberin contents in DT. An auxin-induced process requires the local breaking and resealing of endodermal apoplastic barriers during lateral root emergence in *Arabidopsis* (Ursache et al., 2021). However, our qRT-PCR data did not correspond to the structure and composition results (**Figure 7**). Considering that root apoplastic barriers are both involved in root development and induced by stress responses, the expression of related genes in different zones of the root was very different (Wang et al., 2019). Further detailed investigation is needed to address this.

Although the CS and SL in the root endodermis have been described as tight barriers blocking the non-selective apoplastic transport of solutes and water (Geldner, 2013; Nawrath et al., 2013), the CS or SL might participate in the selective uptake of mineral elements in *Arabidopsis* and rice (Baxter et al., 2009; Barberon et al., 2016; Wang et al., 2019; Cohen et al., 2020; Wang et al., 2022). Suberization also shows nutrient-induced plasticity, and regulated by hormones such as abscisic acid, ethylene, and auxin (Barberon et al., 2016; Ursache et al., 2021). Here, DT plants developed stronger apoplastic barriers and accumulated less Na, Mg, Mn, and Zn and more Ni, Cu, and Al than DS, regardless of osmotic stress (**Figure 6**). Whether this phenomenon is closely associated with the formation of the CS and SL remains unclear, but these results provide ideas for further investigation of the exact roles of apoplastic barriers in the selective uptake of mineral elements in grass plants. Furthermore, the present study found that except for Na and Zn, most of the mineral elements accumulated more in leaves under osmotic stress conditions in both DT and DS (**Figure 6**), suggesting a potential role for endodermal barriers in preventing mineral nutrient reflux to the soil under osmotic stress.

Root morphology and architecture play a pivotal role in plant drought responses (Bengough et al., 2011; Kreszies et al., 2019). Our results suggested that reduced lateral root formation, rather than total seminal root length, differentiates DT from DS when adapting to a water deficit (**Figure 3**). This result differs from the findings of a comparative study of barley and wild barley, which showed that wild barley always had longer seminal roots, regardless of osmotic stress (Kreszies et al., 2020). Furthermore, longer and thicker seminal roots, more lateral roots, and more complex root systems developed in DT than in DS plants. This suggested a genetically fixed developmental trend and a stronger drought response strategy in DT to uptake water in deeper and wider soil areas. Although our experiments proceeded under artificial hydroponic conditions.

## Conclusions

We identified DT and DS *E. sibiricus* genotypes among 52 genotypes based on an evaluation of drought tolerance. The root apoplastic bypass flow of water and solutes, as well as mineral nutrient accumulation differed between DT and DS. In addition, *E. sibiricus* roots did not form an exodermis, endodermal barriers (CS and SL) were more developed, and more suberin monomers were deposited in DT than DS plants. Osmotic stress induced the formation of barriers closer to the root tip in DS, but possibly increased their thickness in DT. Our results suggested that the establishment of a complete apoplastic barrier in the endodermis facilitates drought tolerance of *E. sibiricus*. Apoplastic barriers might also contribute to natural variations in drought tolerance between the studied genotypes. The beneficial traits of DT (I-1-5-46) could be selected for future breeding programs to develop more drought tolerant crops and forage.

## Data availability statement

The original contributions presented in the study are included in the article/**Supplementary Material**. Further inquiries can be directed to the corresponding author.

## Author contributions

PeW conceived and designed the experiments. QZ and PeW provided experimental materials and chemical reagents. XL, PiW, YA, YH, and YZ implemented experiments and analyzed data. PeW and XL drafted the manuscript. C-MW and PeW reviewed and improved the manuscript. All authors have read and approved the published version of the manuscript.

## Funding

This study was supported by grants from the National Natural Science Foundation of China (31802122) and the Fundamental Research Funds for the Central Universities (ZYN2022018).

## Acknowledgments

We thank Wahap Isac (Servicebio) for technical assistance with the histochemical detection of Casparian strips and suberin lamellae. We thank Editage (www.editage.cn) for English language editing.

## Conflict of interest

The authors declare that the research was conducted in the absence of any commercial or financial relationships that could be construed as a potential conflict of interest.

## Publisher’s note

All claims expressed in this article are solely those of the authors and do not necessarily represent those of their affiliated organizations, or those of the publisher, the editors and the reviewers. Any product that may be evaluated in this article, or claim that may be made by its manufacturer, is not guaranteed or endorsed by the publisher.
