# Supplementary material for: Endodermal apoplastic barriers are linked to osmotic tolerance in meso-xerophytic grass Elymus sibiricus
Source: Front Plant Sci. 2022 Sep 23;13:1007494. doi: 10.3389/fpls.2022.1007494 (PMC9539332; doi:10.3389/fpls.2022.1007494)
Supplement: Supplementary file 1 [file DataSheet_1.docx]

**SUPPLEMENTARY TABLE 1. Identity and origin of drought resistance of** **52 *E. sibiricus* genotypes**

| **ID** | **Geographic information** | **Altitude(m)** | **ID** | **Geographic information** | **Altitude(m)** |
| --- | --- | --- | --- | --- | --- |
| 08-129 | 100°35′E, 35°14′N | 3107 | 09-055 | 102°6′E, 36°24′N | 2482 |
| 09-071 | 102°2′E, 36°23′N | 2522 | 09-083 | 102°2′E, 36°23′N | 2522 |
| 09-089 | 102°2′E, 36°23′N | 2522 | 09-124 | 101°56′E, 36°25′N | 2377 |
| 09-149 | 101°9′E, 36°31′N | 3036 | 09-152 | 101°9′E, 36°31′N | 3036 |
| 09-183 | 100°54′E, 36°57′N | 3116 | 09-244 | 97°12′E, 37°18′N | 2895 |
| 09-280 | 91°54′E, 29°45′N | 3959 | 10-5 | 100°14′E, 35°41′N | 2755 |
| 11-14 | 102°45′E, 35°58′N | 2243 | 14-091 | 103°38′E, 32°30′N | 2702 |
| 14-16-2 | 102°58′E, 33°09′N | 3548 | 14-694 | 103°38′E, 32°30′N | 2702 |
| B-12-13-2 | 102°57′E, 32°83′N | 3513 | B-19-9-1 | 102°57′E, 32°83′N | 3513 |
| I-1-3-3 | 100°8′E, 37°19′N | 3297 | I-1-4-1 | 102°57′E, 32°83′N | 3153 |
| I-1-4-12 | 98°49′E, 37°52′N | 3841 | I-1-5-18 | 98°1′E, 38°42′N | 3705 |
| I-1-5-2 | 100°25′E, 35°13′N | 3231 | I-1-5-20 | 100°25′E, 35°13′N | 3231 |
| I-1-5-21 | 100°46′E, 32°55′N | 3488 | I-1-5-23 | 100°25′E, 35°13′N | 3231 |
| I-1-5-25 | 100°35′E, 33°5′N | 3662 | I-1-5-28 | 101°28′E, 35°21′N | 3644 |
| I-1-5-29 | 100°28′E, 35°35′N | 3300 | I-1-5-3 | 100°8′E, 37°19′N | 3297 |
| I-1-5-30 | 98°12′E, 34°55′N | 4276 | I-1-5-39 | 100°48′E, 35°15′N | 3326 |
| I-1-5-40 | 100°23′E, 35°16′N | 2952 | I-1-5-41 | 99°25′E, 33°43′N | 4051 |
| I-1-5-42 | 99°52′E, 34°35′N | 3702 | I-1-5-45 | 101°2′E, 33°45′N | 3627 |
| I-1-5-46 | 99°47′E, 33°37′N | 4025 | I-1-5-49 | 97°30′E, 36°2′N | 2981 |
| I-1-5-50 | 100°8′E, 37°19′N | 3297 | I-1-5-53 | 99°38′E, 36°59′N | 3206 |
| I-1-5-58 | 100°56′E, 35°54′N | 2729 | I-1-5-59 | 113°37′E, 38°56′N | 2043 |
| I-1-5-60 | 81°16′E, 43°54′N | 631 | I-1-5-61 | 88°6′E, 43°53′N | 1790 |
| I-1-5-63 | 87°10′E, 43°30′N | 1172 | I-1-5-66 | 81°6′E, 42°35′N | 2233 |
| I-1-5-67 | 115°34′E, 39°46′N | 1478 | I-1-5-71 | 101°9′E, 36°31′N | 3036 |
| I-1-6-2 | 116°48′E, 43°42′N | 1274 | I-5-14 | 99°39′E, 33°59′N | 3995 |
| I-5-2-9 | 100°20′E, 35°3′N | 3295 | SWUN 2 | 102°57′E, 32°83′N | 3513 |

**SUPPLEMENTARY TABLE 2. Primers used for qRT-PCR**

| **Primer name** | **Sequence (5'to3')** | **Annealing temperature (℃)** |
| --- | --- | --- |
| **DNAJ-F** | GCAATGGCGTCAATGGCTTCAC | 58 |
| **DNAJ-R** | GCATCACTAAGTCTGGACACCTCAG |  |
| **MYB41-F** | GGCTCATCATCACCCTCCA | 60 |
| **MYB41-R** | TGTGCGTGTTCCAGTAGTTCTT |  |
| **CYP86A1-F** | AAGATGTCGCTCACTCTGTTCA | 60 |
| **CYP86A1-R** | GGCAGTGGTGGTTGGGAT |  |
| **KCS20-F** | TCACCCATCAAACGAAGC | 58 |
| **KCS20-R** | ATGAAACGACGCACTCCA |  |
| **FAR1-F** | GTTTCGGTATCCCCTTTCG | 58 |
| **FAR1-R** | GCACATCCGCATACAATCTC |  |
| **SHR1-F** | ACGCTTCTTCTCGGCTTACA | 58 |
| **SHR1-R** | CGCCCTGCTCCCCTCT |  |
| **MYB36-F** | CACCTTTTACGGCAACCT | 55 |
| **MYB36-R** | TGGATATGAGACCAGAACCC |  |
| **PER64-F** | AGAAACACGGCGGAGAA | 55 |
| **PER64-R** | CGTTGTGGACGACGTAGAA |  |
| **CASP -F** | TACCAAGAGGAAGTTGACAATC | 55 |
| **CASP -R** | CAGCCATAGAAGCAAGAGC |  |

**SUPPLEMENTARY TABLE 3. Coefficient of variation of 13 physiological traits between 52 *E. sibiricus* genotypes**

| **Parameter** | **Physiological indices** | | | | | | | | | | | | |
| --- | --- | --- | --- | --- | --- | --- | --- | --- | --- | --- | --- | --- | --- |
|  | **Chl a** | **Chl b** | **Car** | **REC** | **MDA** | **Fo** | **Fm** | **Fv/Fm** | **NPQ** | **ETR** | **qP** | **ΦPSⅡ** | **Fv'/Fm'** |
| **Control** | | | | | | | | | | | | | |
| **Maximum value** | 37.46 | 17.30 | 5.71 | 0.75 | 26.96 | 224.60 | 960.13 | 0.82 | 0.63 | 29.56 | 0.75 | 0.79 | 0.89 |
| **Minimum**  **value** | 7.11 | 2.86 | 0.45 | 0.01 | 1.10 | 56.51 | 305.27 | 0.73 | 0.05 | 7.55 | 0.30 | 0.35 | 0.54 |
| **Mean Value** | 16.75 | 6.25 | 3.11 | 0.66 | 5.54 | 125.77 | 603.03 | 0.79 | 0.39 | 15.72 | 0.47 | 0.46 | 0.68 |
| **Standard Deviation** | 5.56 | 2.59 | 1.18 | 0.09 | 3.92 | 38.09 | 177.78 | 0.02 | 0.16 | 4.45 | 0.08 | 0.08 | 0.08 |
| **CV** | 33.17 | 41.49 | 37.78 | 13.67 | 70.79 | 30.28 | 29.48 | 1.96 | 42.66 | 28.33 | 16.40 | 16.39 | 11.07 |
| **Drought Stress** | | | | | | | | | | | | | |
| **Maximum value** | 25.50 | 8.64 | 4.92 | 0.97 | 83.05 | 277.44 | 728.55 | 0.79 | 0.82 | 17.67 | 0.55 | 0.54 | 0.75 |
| **Minimum value** | 4.04 | 0.80 | 0.39 | 0.03 | 11.99 | 68.96 | 167.00 | 0.17 | 0.11 | 3.19 | 0.06 | 0.11 | 0.17 |
| **Mean Value** | 9.39 | 3.30 | 1.98 | 0.62 | 37.96 | 151.72 | 372.74 | 0.58 | 0.54 | 9.33 | 0.33 | 0.33 | 0.46 |
| **Standard Deviation** | 4.50 | 1.57 | 0.87 | 0.26 | 16.44 | 48.58 | 114.00 | 0.13 | 0.20 | 3.91 | 0.09 | 0.07 | 0.11 |
| **CV** | 47.95 | 47.74 | 43.99 | 41.57 | 43.30 | 32.02 | 30.58 | 22.22 | 36.53 | 41.88 | 26.44 | 22.44 | 24.59 |
| **CV_D_** | 0.36 | 0.14 | 0.15 | 1.01 | 0.48 | 0.06 | 0.04 | 1.68 | 0.16 | 0.39 | 0.47 | 0.31 | 0.76 |

Note: Chl a: chlorophyll a; Chl b: chlorophyll b; Car: carotenoid; REC: Relatively conductivity rate; MDA: malonaldehyde content; Fo: minimal fluorescence; Fm: maximal fluorescence; Fv/Fm: PS Ⅱ maximum photochemical quantum yield; NPC: non-photochemical quenching coefficient; ETR: electron transport rate; qP: photochemical quenching coefficient; ΦPSⅡ: quantum yield of PSⅡelectron transport; Fv'/ Fm': PSII effective photochemistry quanta output; CV: coefficient of variation; CV_D_: coefficient of variation for drought.

**SUPPLEMENTARY TABLE 4. Relative value of 13 traits between 52 *E. sibiricus* genotypes**

|  | **Trait relative value** | | | | | | | | | | | | |
| --- | --- | --- | --- | --- | --- | --- | --- | --- | --- | --- | --- | --- | --- |
| **ID** | **Chl a** | **Chl b** | **Car** | **REC** | **MDA** | **Fo** | **Fm** | **Fv/Fm** | **NPQ** | **ETR** | **qP** | **ΦPSⅡ** | **Fv'/ Fm'** |
| **08-129** | 0.71 | 0.61 | 0.75 | 7.50 | 9.98 | 1.05 | 0.68 | 0.86 | 1.45 | 0.80 | 0.81 | 0.67 | 0.74 |
| **09-055** | 0.38 | 0.36 | 0.75 | 4.38 | 7.82 | 1.21 | 0.79 | 0.87 | 1.29 | 0.67 | 0.67 | 0.73 | 0.89 |
| **09-071** | 0.30 | 0.31 | 0.31 | 2.34 | 8.88 | 1.10 | 0.69 | 0.84 | 1.56 | 0.34 | 0.82 | 0.67 | 0.77 |
| **09-083** | 0.64 | 0.43 | 0.68 | 30.44 | 7.95 | 1.15 | 0.55 | 0.72 | 1.29 | 0.45 | 0.80 | 0.69 | 0.84 |
| **09-089** | 0.92 | 0.90 | 0.84 | 23.99 | 8.10 | 2.06 | 0.55 | 0.32 | 2.23 | 0.49 | 0.80 | 0.72 | 0.67 |
| **09-124** | 0.59 | 0.79 | 0.65 | 33.53 | 7.22 | 1.83 | 0.61 | 0.47 | 1.32 | 0.45 | 0.80 | 0.79 | 0.66 |
| **09-149** | 0.34 | 0.32 | 0.74 | 5.51 | 7.49 | 2.09 | 0.72 | 0.48 | 1.35 | 0.80 | 0.75 | 0.71 | 0.70 |
| **09-152** | 0.78 | 0.38 | 0.86 | 2.35 | 6.92 | 1.21 | 0.70 | 0.80 | 1.27 | 0.67 | 0.73 | 0.75 | 0.90 |
| **09-183** | 0.79 | 0.51 | 0.81 | 1.78 | 6.95 | 1.02 | 0.63 | 0.83 | 1.33 | 0.34 | 0.75 | 0.70 | 0.72 |
| **09-244** | 0.42 | 0.70 | 0.94 | 11.06 | 7.39 | 1.09 | 0.66 | 0.83 | 1.55 | 0.45 | 0.83 | 0.73 | 0.87 |
| **09-280** | 0.82 | 0.93 | 0.88 | 21.28 | 7.85 | 1.06 | 0.73 | 0.89 | 1.26 | 0.49 | 0.75 | 0.68 | 0.64 |
| **10-5** | 0.90 | 0.94 | 0.80 | 10.59 | 7.22 | 1.18 | 0.60 | 0.74 | 1.38 | 0.45 | 0.83 | 0.72 | 0.72 |
| **11-14** | 0.62 | 0.39 | 0.85 | 18.99 | 8.59 | 1.14 | 0.52 | 0.68 | 1.28 | 0.86 | 0.69 | 0.73 | 0.64 |
| **14-091** | 0.90 | 0.82 | 0.78 | 4.41 | 7.94 | 1.28 | 0.63 | 0.73 | 1.28 | 0.64 | 0.75 | 0.78 | 0.98 |
| **14-16-2** | 0.52 | 0.41 | 0.92 | 7.35 | 7.85 | 1.47 | 0.76 | 0.73 | 1.35 | 0.47 | 0.70 | 0.72 | 0.65 |
| **14-694** | 0.65 | 0.59 | 0.77 | 47.43 | 10.60 | 1.44 | 0.61 | 0.65 | 1.44 | 0.42 | 0.90 | 0.76 | 0.63 |
| **B-12-13-2** | 0.39 | 0.45 | 0.72 | 12.17 | 11.51 | 1.20 | 0.73 | 0.81 | 1.59 | 0.58 | 0.69 | 0.57 | 0.56 |
| **B-12-9-1** | 0.92 | 0.90 | 0.95 | 1.99 | 2.05 | 1.24 | 0.94 | 0.93 | 1.12 | 0.94 | 0.94 | 0.97 | 0.94 |
| **I-1-3-3** | 0.44 | 0.26 | 0.50 | 35.09 | 8.58 | 1.05 | 0.67 | 0.87 | 1.29 | 0.75 | 0.83 | 0.56 | 0.77 |
| **I-1-4-1** | 0.68 | 0.65 | 0.50 | 18.26 | 9.88 | 1.14 | 0.64 | 0.79 | 1.33 | 0.71 | 0.63 | 0.62 | 0.82 |
| **I-1-4-12** | 0.47 | 0.68 | 0.62 | 12.00 | 9.20 | 1.03 | 0.97 | 0.98 | 1.28 | 0.31 | 0.84 | 0.64 | 0.80 |
| **I-1-5-18** | 0.39 | 0.35 | 0.66 | 10.32 | 7.77 | 1.34 | 0.65 | 0.74 | 1.50 | 0.52 | 0.74 | 0.73 | 0.76 |
| **I-1-5-2** | 0.28 | 0.30 | 0.26 | 19.92 | 17.22 | 1.03 | 0.28 | 0.27 | 3.71 | 0.18 | 0.16 | 0.36 | 0.18 |
| **I-1-5-20** | 0.31 | 0.30 | 0.52 | 15.71 | 7.46 | 1.18 | 0.79 | 0.88 | 1.29 | 0.43 | 0.70 | 0.69 | 0.80 |
| **I-1-5-21** | 0.29 | 0.56 | 0.73 | 8.48 | 10.34 | 1.52 | 0.66 | 0.69 | 1.27 | 0.96 | 0.64 | 0.60 | 0.81 |
| **I-1-5-23** | 0.87 | 0.85 | 0.84 | 15.14 | 10.32 | 1.29 | 0.63 | 0.74 | 1.30 | 0.60 | 0.56 | 0.66 | 0.81 |
| **I-1-5-25** | 0.77 | 0.86 | 0.59 | 14.05 | 9.54 | 1.07 | 0.71 | 0.87 | 1.28 | 0.57 | 0.90 | 0.59 | 0.77 |
| **I-1-5-28** | 0.66 | 0.43 | 0.56 | 28.49 | 9.72 | 1.36 | 0.74 | 0.78 | 1.29 | 0.38 | 0.70 | 0.63 | 0.73 |
| **I-1-5-29** | 0.42 | 0.41 | 0.41 | 5.62 | 8.77 | 1.15 | 0.64 | 0.82 | 1.50 | 0.71 | 0.64 | 0.64 | 0.73 |
| **I-1-5-3** | 0.29 | 0.32 | 0.32 | 17.76 | 16.24 | 1.09 | 0.31 | 0.22 | 3.81 | 0.23 | 0.30 | 0.31 | 0.16 |
| **I-1-5-30** | 0.70 | 0.49 | 0.62 | 6.65 | 6.98 | 1.10 | 0.49 | 0.72 | 1.51 | 0.69 | 0.66 | 0.64 | 0.61 |
| **I-1-5-39** | 0.64 | 0.59 | 0.92 | 5.38 | 6.94 | 1.08 | 0.53 | 0.74 | 1.30 | 0.78 | 0.79 | 0.68 | 0.68 |
| **I-1-5-40** | 0.35 | 0.24 | 0.80 | 30.47 | 7.00 | 1.11 | 0.55 | 0.74 | 1.27 | 0.59 | 0.66 | 0.67 | 0.62 |
| **I-1-5-41** | 0.26 | 0.14 | 0.40 | 17.54 | 9.09 | 1.18 | 0.69 | 0.82 | 1.37 | 0.75 | 0.86 | 0.62 | 0.94 |
| **I-1-5-42** | 0.93 | 0.79 | 0.66 | 17.19 | 6.94 | 1.10 | 0.54 | 0.74 | 1.32 | 0.55 | 0.66 | 0.67 | 0.55 |
| **I-1-5-45** | 0.68 | 0.97 | 0.62 | 36.14 | 7.07 | 1.18 | 0.61 | 0.74 | 1.55 | 0.71 | 0.69 | 0.67 | 0.79 |
| **I-1-5-46** | 0.94 | 0.82 | 0.92 | 1.94 | 2.05 | 1.13 | 0.96 | 0.95 | 1.12 | 0.93 | 0.94 | 0.98 | 0.93 |
| **I-1-5-49** | 0.95 | 0.62 | 0.62 | 6.52 | 6.71 | 1.09 | 0.65 | 0.84 | 1.29 | 0.57 | 0.62 | 0.65 | 0.89 |
| **I-1-5-50** | 0.44 | 0.33 | 0.63 | 5.06 | 6.73 | 1.07 | 0.60 | 0.79 | 1.58 | 0.68 | 0.78 | 0.71 | 0.74 |
| **I-1-5-53** | 0.39 | 0.40 | 0.35 | 30.47 | 12.92 | 1.11 | 0.34 | 0.34 | 4.33 | 0.28 | 0.31 | 0.28 | 0.36 |
| **Table S2 (continued)** | | | | | | | | | | | | | |
| **I-1-5-58** | 0.24 | 0.18 | 0.56 | 1.22 | 6.72 | 1.01 | 0.52 | 0.74 | 1.30 | 0.47 | 0.57 | 0.68 | 0.45 |
| **I-1-5-59** | 0.31 | 0.16 | 0.39 | 2.55 | 6.80 | 1.03 | 0.55 | 0.77 | 1.26 | 0.60 | 0.76 | 0.64 | 0.74 |
| **I-1-5-60** | 0.97 | 0.97 | 0.51 | 2.15 | 7.37 | 1.05 | 0.49 | 0.68 | 1.30 | 0.65 | 0.72 | 0.68 | 0.67 |
| **I-1-5-61** | 0.55 | 0.86 | 0.50 | 23.39 | 7.28 | 1.05 | 0.58 | 0.78 | 1.84 | 0.83 | 0.69 | 0.67 | 0.87 |
| **I-1-5-63** | 0.92 | 0.90 | 0.92 | 1.82 | 2.49 | 1.17 | 0.93 | 0.91 | 1.10 | 0.92 | 0.93 | 0.96 | 0.95 |
| **I-1-5-66** | 0.80 | 0.49 | 0.91 | 1.35 | 4.31 | 1.06 | 0.47 | 0.69 | 1.89 | 0.44 | 0.65 | 0.72 | 0.66 |
| **I-1-5-67** | 0.47 | 0.45 | 0.65 | 9.67 | 6.84 | 1.77 | 0.74 | 0.65 | 1.29 | 0.73 | 0.81 | 0.71 | 0.69 |
| **I-1-5-71** | 0.26 | 0.51 | 0.65 | 22.79 | 4.40 | 1.25 | 0.52 | 0.65 | 1.35 | 0.38 | 0.83 | 0.69 | 0.70 |
| **I-1-6-2** | 0.30 | 0.39 | 0.34 | 18.70 | 10.03 | 1.15 | 0.63 | 0.72 | 1.27 | 0.80 | 0.70 | 0.70 | 0.76 |
| **I-5-14** | 0.56 | 0.73 | 0.60 | 2.45 | 7.18 | 1.51 | 0.76 | 0.73 | 1.31 | 0.71 | 0.68 | 0.68 | 0.66 |
| **I-5-2-9** | 0.90 | 0.67 | 0.60 | 23.95 | 8.10 | 1.06 | 0.46 | 0.69 | 2.01 | 0.56 | 0.69 | 0.69 | 0.80 |
| **SWUN 2** | 0.63 | 0.54 | 0.93 | 25.16 | 4.82 | 1.26 | 0.65 | 0.74 | 1.50 | 0.80 | 0.63 | 0.66 | 0.58 |

Note: Chl a: chlorophyll a; Chl b: chlorophyll b; Car: carotenoid; REC: Relatively conductivity rate; MDA: malonaldehyde content; Fo: minimal fluorescence; Fm: maximal fluorescence; Fv/Fm: PSⅡ maximum photochemical quantum yield; NPC: non-photochemical quenching coefficient; ETR: electron transport rate; qP: photochemical quenching coefficient; ΦPSⅡ: quantum yield of PSⅡ electron transport; Fv'/ Fm': PSII effective photochemistry quanta output.

**SUPPLEMENTARY TABLE 5. Mineral elemental concentrations in shoots of DT (drought tolerant genotype) and DS (drought sensitive genotype) grown under control or osmotic stress condition.**

| **Element** | **DS-control**  **(μg⋅g^-1^ DW)** | **DS-20%PEG**  **(μg⋅g^-1^ DW)** | **DT-control**  **(μg⋅g^-1^ DW)** | **DT-20%PEG**  **(μg⋅g^-1^ DW)** |
| --- | --- | --- | --- | --- |
| **B** | 13.55±0.49b | 16.96±1.01a | 11.31±0.93c | 15.75±0.10a |
| **Na** | 10895.31±1017.25a | 9976.16±770.10ab | 8765.69±866.71b | 6628.66±721.47c |
| **Mg** | 2461.53±78.88b | 2749.21±48.33a | 1981.66±42.76d | 2271.11±82.69c |
| **K** | 41628.67±971.20b | 44209.59±337.56a | 39522.41±431.71c | 44524.59±803.88a |
| **Ca** | 4578.78±78.79c | 6163.19±326.80a | 4309.19±103.70c | 5293.75±123.69b |
| **Mn** | 98.68±2.22b | 116.64±2.00a | 74.18±2.06c | 81.98±2.44c |
| **Fe** | 162.31±4.73b | 203.84±6.83a | 167.06±7.18b | 198.05±5.36a |
| **Ni** | 1.46±0.02c | 1.56±0.10c | 1.76±0.13b | 2.42±0.14a |
| **Cu** | 22.00±2.71c | 30.07±1.42b | 26.37±1.66b | 37.43±2.71a |
| **Zn** | 48.54±1.51a | 44.57±2.41b | 34.49±2.47c | 33.57±1.62c |
| **Al** | 50.17±2.22c | 55.16±1.70c | 116.86±10.25b | 411.26±26.11a |

Note: Values are means ± standard deviation (SD) (n =5). Different letters indicate significant differences at *P<0.05* in one‐way analysis of variance (Fishers Duncan test). DW: Dry weight.


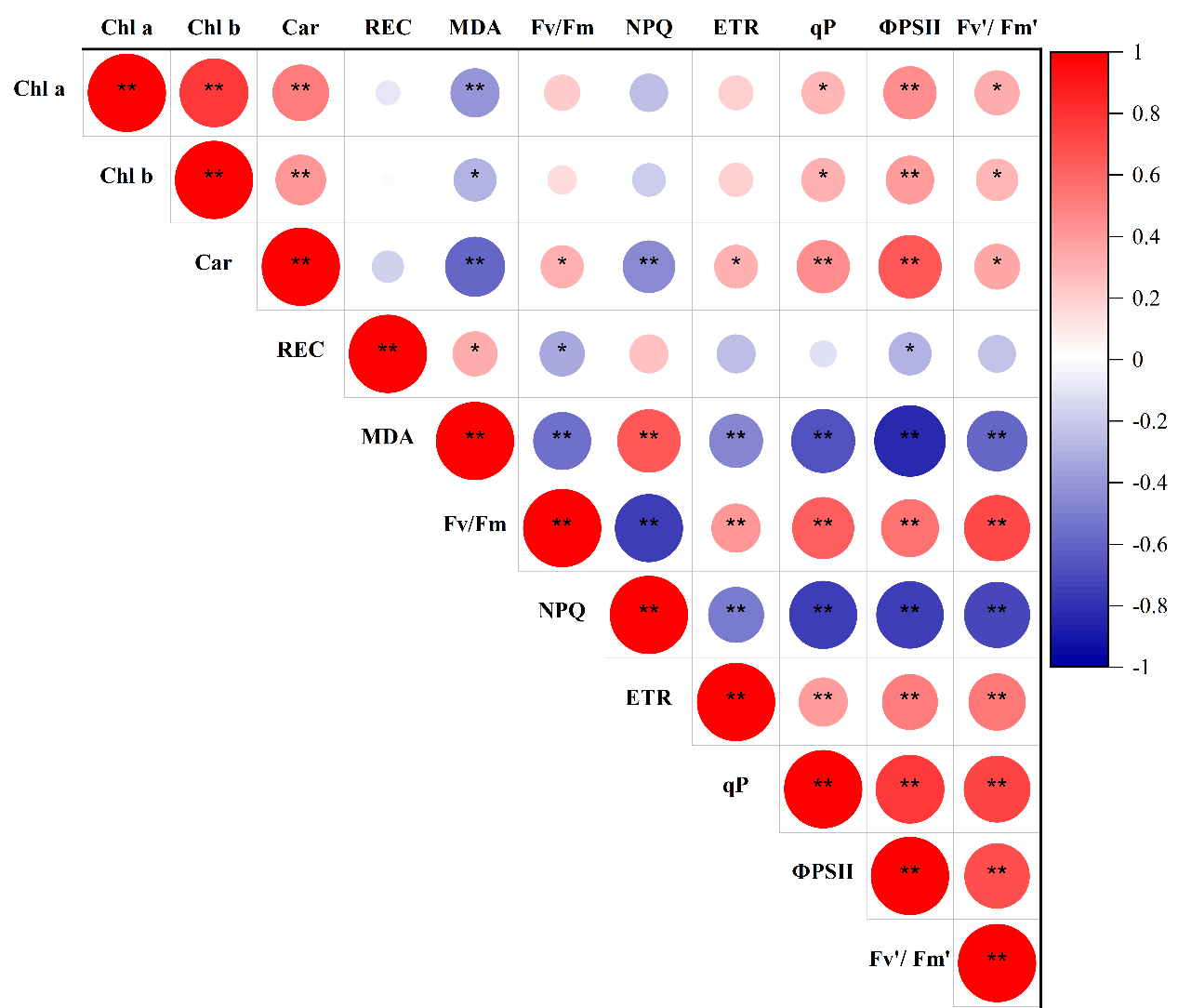


**SUPPLEMENTARY FIGURE 1. Pearson correlation coefficients of physiological traits among 52 *E. sibiricus* genotypes under drought stress.** Pearson correlation coefficients were conducted based on the data for **Supplementary Table 2**. The circle size and shade correspond to the absolute value, with red indicating a positive correlation and blue indicating a negative correlation. * and ** mean significant correlation at 0.05 and 0.01 level, respectively. Chl a: chlorophyll a; Chl b: chlorophyll b; Car: carotenoid; REC: Relatively conductivity rate; MDA: malonaldehyde content; Fv/Fm: PSⅡmaximum photochemical quantum yield; NPC: non-photochemical quenching coefficient; ETR: electron transport rate; qP: photochemical quenching coefficient; ΦPSⅡ: quantum yield of PSⅡ electron transport; Fv'/ Fm': PSII effective photochemistry quanta output.


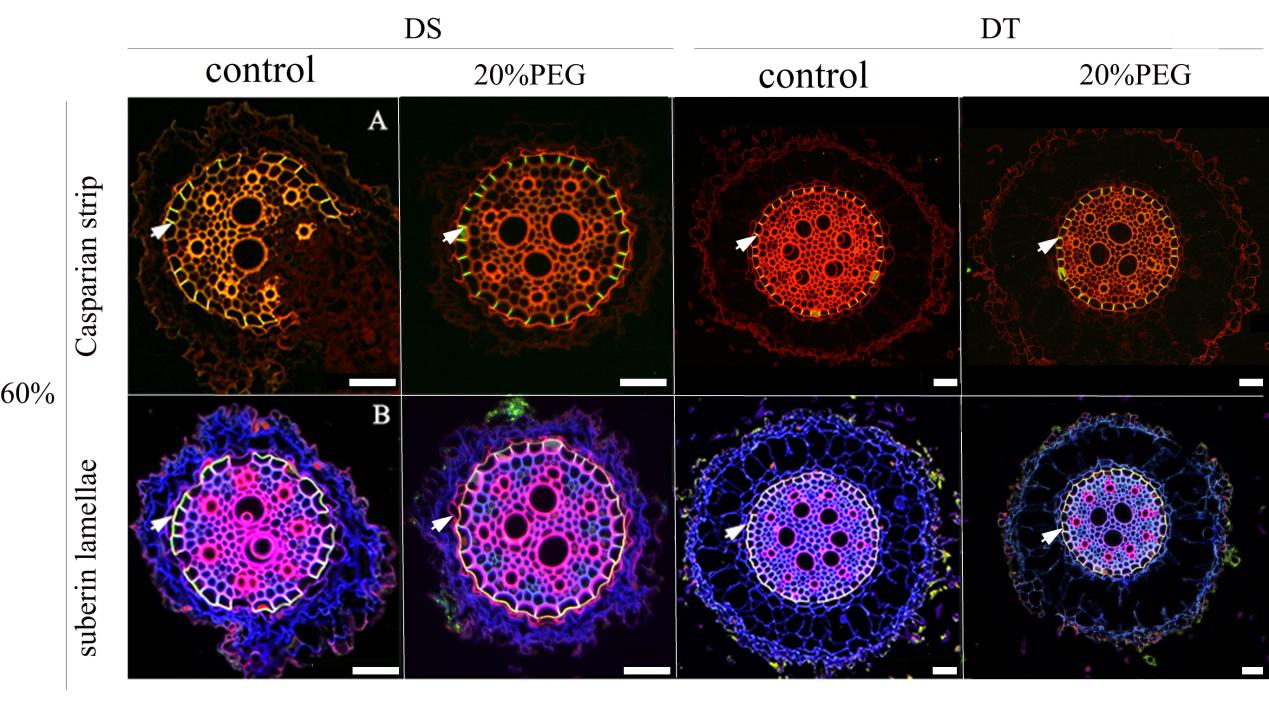


**SUPPLEMENTARY FIGURE 2. Fluorescence staining of apoplastic barriers in seminal roots of DT (drought tolerant genotype) and DS (drought sensitive genotype) grown under control and osmotic stress condition**. **(A)** The Casparian strip (CS) were stained with berberine hemisulfate at 60% from the tip, and green fluorescence (white arrow) indicated that the CS was present only in the endodermis but not in the exodermis. **(B)** The suberin lamella (SL) were stained with fluorescent yellow 088 at 60% from the tip of DT and DS, yellow fluorescence (white arrow) indicated that the CS was present only in the endodermis but not in the exodermis. (Scale bars, 42.5 µm).
